# Tripartite factors leading to molecular divergence between human and murine smooth muscle

**DOI:** 10.1371/journal.pone.0227672

**Published:** 2020-01-16

**Authors:** Soo Jung Lee, Sabrina Blanchett-Anderson, Simon G. Keep, Mitchell B. Gasche, Michael M. Wang

**Affiliations:** 1 Department of Neurology, University of Michigan, Ann Arbor, Michigan, United States of America; 2 Neurology Service, VA Ann Arbor Healthcare System, Ann Arbor, Michigan, United States of America; 3 Department of Molecular & Integrative Physiology, University of Michigan, Ann Arbor, Michigan, United States of America; National Center for Toxicological Research, UNITED STATES

## Abstract

A large number of pre-clinical and developmental investigations involve experimental vertebrate animals, of which mice have emerged as a favored organism. Recognition of the differences between humans and mice is essential for assessment of the relevance of animal studies to humans. The primary purpose of this study was to gauge the conservation between human and mouse vascular smooth muscle cell (VSMC) proteins mined from an analysis of the Human Protein Atlas. Two comparison were made: a) immunohistochemistry for 16 proteins in brain, heart, esophagus, bladder, stomach, lung, kidney, and aorta enabled comparison between human and mouse of protein localization in VSMC and non-vascular SMC; and b) multi-species primary protein sequence analysis of an expanded set vascular molecules enabled comparison between VSMC sequences among vertebrate species. In total, three dimensions of diversity were uncovered. First, a significant number of factors show human/mouse differences in cellular expression; these differences occurred in both VSMC and non-vascular SMC in an organ and cell-type dependent fashion. Many markers demonstrated notable cell-to-cell and regional heterogeneity in VSMC of the aorta and non-vascular SMC of the esophagus, bladder, and stomach. Second, species specificity can arise by genetic deletions as exemplified by the human protein adipogenesis regulatory factor (ADIRF), which is not present due to a large sequence gap in mice. Third, we describe significant cross-species protein sequence divergence in selected VSMC proteins which may result in altered orthologue function. In a sample of 346 vascular molecules, 15% demonstrate incomplete vertebrate species gene conservation. Divergence of predicted human/mouse VSMC protein sequences is higher than for endothelial proteins in all species examined. In the future, each of these three cross-species differences could be neutralized using gene manipulation, resulting in improved translational potential of murine experimental models.

## Introduction

The importance of the vascular system in physiology of all organs and in human disease has driven efforts to understand blood vessels at the molecular level. For example, endothelial cell (EC) expression profiles have been described in detail on a global basis in numerous transcriptome and proteome wide efforts [1–4]. However, similar in depth understanding of proteins in vascular smooth muscle cells (VSMC) is less well-developed. This knowledge-gap prompted a recent study of global protein expression in humans that gave equal emphasis to brain VSMC and EC proteins and resulted in identification of a panel of new VSMC molecules in brain [3]. The functions of these newly identified VSMC proteins remain largely unknown, but the scope of this endeavor requires additional characterization to enable prioritization of future functional analysis.

Current translational studies rely heavily on mouse models of disease that enable delineation of molecular mechanism. However, many studies of vascular diseases have failed to demonstrate clinical efficacy of treatments that proved effective in mice and other model organisms. For example, in cerebrovascular disease, human clinical trials have not succeeded using agents validated in mouse models [5–7]. Furthermore, CADASIL, the most common inherited cause of stroke and vascular dementia and a result of failure of VSMC, is not recapitulated in mice harboring gene mutations found in patients [8–10]. In other fields as well, only a minority of mouse studies yield successful human clinical applications; in cancer, the translational success rate from mouse to human is 10% [11]. In gastrointestinal disorders, drug screening for anti-gastrosecretory drugs using rodents led to agents that were ineffective in people [12].

The challenges of building bridges that connect mouse models to human pathology suggest potential dissimilarities between mouse and human blood vessels. Transcriptome analysis has demonstrated divergence between mouse and human RNA expression patterns in tissues and organs [13]; however, little is known at cellular resolution, and few studies focus on protein differences. Several recent studies suggest molecular differences between human and mouse EC protein expression patterns [14, 15]. But the molecular differences between VSMC of humans and mice have not been addressed.

We hypothesized that human and mouse VSMC protein localization and sequence are incompletely conserved. To test this, we examined 16 human VSMC proteins which were discovered in an analysis of the Human Protein Atlas as having reliable SMC staining. We uncover examples of discordance of immunohistochemical (IHC) expression patterns between mouse and human organs in both VSMC and non-vascular SMC. We also identified non-conserved vertebrate VSMC protein-encoding genes and quantified the level of SMC protein sequence conservation across multiple species.

## Methods

### Animal studies

This study used vertebrate animals. All experiments that involved animals were performed in accordance with NIH guidelines. Experiments were performed using protocols approved by the Institutional Animal Use and Care Committee (IACUC) at the University of Michigan and the VA Ann Arbor Healthcare System. Mice used in the study were provided food and water ad libitum and maintained in enriched, temperature controlled environments under 12 hours light and 12 hour dark cycles. All animals were euthanized in a carbon dioxide chamber which was approved by the IACUC; decapitation was used as a second confirmatory method of sacrifice. Tissues used for protein analysis were from FVB/N mice (all proteins examined) and confirmatory studies were performed on C57/BL6 mice (see S6 Fig); strains originated from colonies maintained at the VA Ann Arbor.

### Immunohistochemistry

To confirm staining patterns from the Human Protein Atlas [16], we examined formalin fixed frontal lobes sections obtained from the Alzheimer’s Disease Center at the University of Michigan and the Brain Bank of the National Institute for Developmental and Childhood Disorders at the University of Maryland. For mouse studies, we studied formalin fixed tissues from multiple organs that were processed in an identical protocol to our human samples. Five micron sections were analyzed using chromogenic immunohistochemical staining using antibodies after citrate-induced antigen retrieval [15, 17, 18]. Staining was followed by hematoxylin counterstaining. Human brain vessel protein integrity was confirmed by staining with the monoclonal antibody BRIC231 (anti-H; Santa Cruz). Integrity of mouse tissue antigens was uniformly satisfactory as multiple antibodies resulted in staining patterns that closely resembled expected vascular distributions seen in humans.

Antibodies used for immunohistochemistry were purchased from Sigma: ADIRF (Sigma cat# HPA026810), AGXT (Sigma cat# HPA035371), DES (Sigma cat# HPA018803), EPX (Sigma cat# HPA050507), FAM124A (Sigma cat# HPA048182), GPX8 (Sigma cat# HPA036720), GZMM (Sigma cat# HPA015624), LRRC41 (Sigma cat# HPA051941), MTFR1 (Sigma cat# HPA023152), NEURL4 (Sigma cat# HPA055314), PRMT2 (Sigma cat# HPA018976), ST6GALNAC6 (Sigma cat# HPA018890), TBC1D2B (Sigma cat# HPA052663), and TEX261 (Sigma cat# HPA016631). There were two exceptions. AOC3 (Sigma cat# HPA000980) did not react to recombinant mouse protein or any mouse tissue; therefore, independent reagents for AOC3 were employed (LSBio cat# LS-B13865-50). Also, the antibodies used in the HPA for LPHN2/ADGRL2 showed weak staining in mouse tissues and the product was discontinued; therefore, independent reagents for ADGRL2 were employed to confirm results (LSBio).

### Western blotting

Western blots were performed using standard methods on nitrocellulose membranes using the same antibodies used for IHC. CNN1 (Sigma cat# HPA014263) was used as an established canonical SMC protein marker. Detection was performed using goat anti-rabbit IRDye 800CW (Li-COR #926–32211) secondary antibody detected by a Li-COR Odyssey CLx imager.

### Data analysis

Staining shown is representative across at least two independent rounds of immunohistochemistry. Scoring of tissue staining was performed by investigators blinded to the tissue distributions observed in human tissues. We report only human/mouse IHC differences which were independently noted by all investigators.

### Identification of non-uniformly conserved proteins

The following informatic search was performed to identify potential vascular genes that are not completely conserved across vertebrate species. A total of 346 vascular genes from all the tables and figures of the study by Lee et al [3] were scored for their presence in different species. We looked at five different databases overall to determine which proteins were incompletely conserved. We initially used three different databases (OrthoDB, PubMed, and GeneCards) to determine if each of the 346 vascular genes was present in a set of vertebrate species (in S1 Fig). We identified absences as significant when all three databases agreed. We could not rely on the use of a single database because of significant disagreement between the sources. For example, when we checked between the presence of genes in 59 species, determined to be experimentally relevant from GeneCards, it was common for OrthoDB to show presence of a gene when PubMed did not. There were marked omissions of genes in cats, pigs, and orangutan in the GeneCards data. A small number of genes were present in the GeneCards analysis, but not in the OrthoDB or PubMed databases. After analysis of all three databases, we compiled a list of 54 genes which were not present in at least one vertebrate species from all three databases. For genes not found in mouse, rat, pig, cow, dog, cat, chimpanzee, orangutan, opossum, or platypus, we performed a second stage verification by searching both BLASTP and Ensembl for the encoded protein to confirm absence from these databases. If a protein was found with over 90% identity using BLASTP and over 70% similarity using Ensembl, it was considered present. When a protein shows less than 70% similarity using Ensembl, we identify them as no close orthologue in species. But when no orthologue was present in a species, we considered it as absent and indicated this with N/A.

### Orthologue sequence conservation analysis

When using Ensembl to generate data for the heat map homology representation and homology grids in S2 Fig, there were discrepancies noted depending on which species was used for the query. To maintain consistency, we used percentage identity reported in Ensembl (labeled as the Target %id) where the species listed on different rows of heat maps were used as query species; for each protein, the queried species identity to other species is arrayed horizontally in heat maps. This resulted in cases when orthologues were identified between two species only when one species was used as the query. For example, when searching for ULBP2, some species (mouse, pig, dog, cow, and opossum) have no orthologue but an orthologue was identified when human, chimpanzee, and orangutan were used as query. When orthologous genes in the target species were assigned a name different from the query species, we reported percentage identity even though the annotated names did not match. For example, when using human as the query for MAS1L, the nine other species except orangutan showed no orthologue. But using the rat as a query, an orthologue was identified in opossum under a name different from MAS1L.

But for heat map homology representation for 16 proteins analyzed by IHC, we used a more inclusive approach to avoid underestimation of sequence homology. As before, when searching for homologous genes in target species, we reported the highest identity percentage found, regardless of whether the annotated name of the gene was identical to the query. In addition, we used the highest identity protein from this search as “query” for the rest of the target species. For example, when using human as the query for DES, the orthologue for orangutan (ENSPPYG00000029437) shows 100% target identity. But when using orangutan as the query for DES, Ensembl shows many results with different annotated names. For this case, we used ENSPPYG00000029437 as query against target species.

After further analysis, it was determined that each of the databases used have been updated since the original dates of scoring, which resulted in minor differences in reporting of homology over time. The results of OrthoDB correspond to dates between October 2017 and December 2017. Results of PubMed correspond to dates between December 2017 and January 2018. Results of GeneCards correspond to dates between January 2018-March 2018. Results of Blast correspond to dates between April 2018- April 2019. Results of Ensembl correspond to dates between October 2018-March 2019.

## Results

The study was designed to test the hypothesis that the cellular expression pattern of human and mouse SMC proteins is not completely conserved between the species. In addition, we quantified the level of protein sequence conservation between SMC proteins across vertebrate species. The study was confined to the analysis of novel sets of vascular proteins that were identified using the Human Protein Atlas in prior work [3].

### Selection of VSMC proteins for cellular localization studies

**Fig 1:**
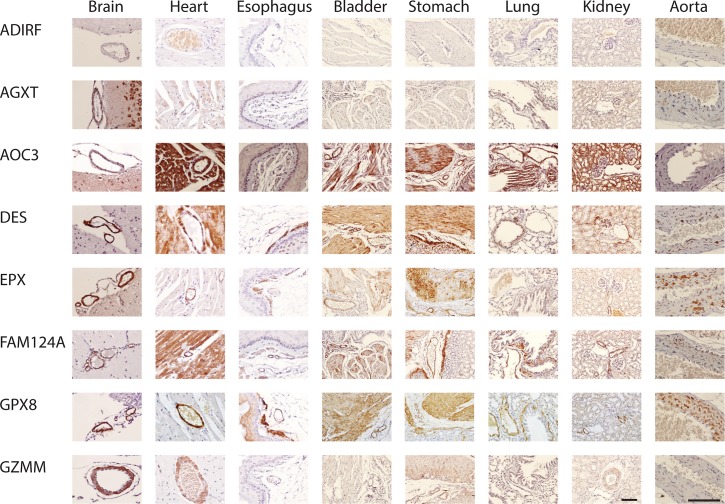
Smooth muscle protein expression in mouse tissues. All antibodies against listed proteins were first validated on human brain as localizing to cerebral vascular smooth muscle. The same antibodies were used on mouse tissues shown here. With few exceptions, protein was localized to mouse cerebral vascular smooth muscle (first column on the left) and to both vascular and non-vascular smooth muscle in other tissues. In lung, non-vascular smooth muscle around the airways was analyzed. In bladder and stomach, non-vascular smooth muscle is shown in the same field as arteries to serve as a comparison for staining intensity on both cell types. The brain, heart, and aorta were viewed at 400X and all other tissues were viewed at 200X; the scale bar is 100 microns for all tissues.

**Fig 2:**
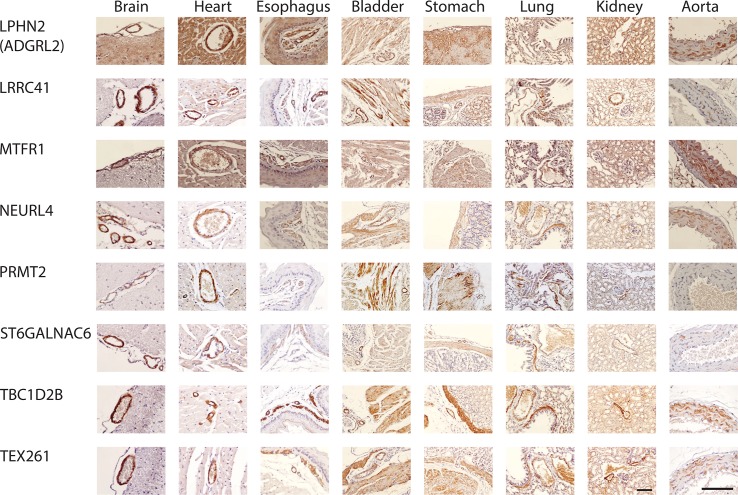
Smooth muscle protein expression in mouse tissues. Additional staining was performed using antibodies generated against proteins listed, as in Fig 1.

**Fig 3:**
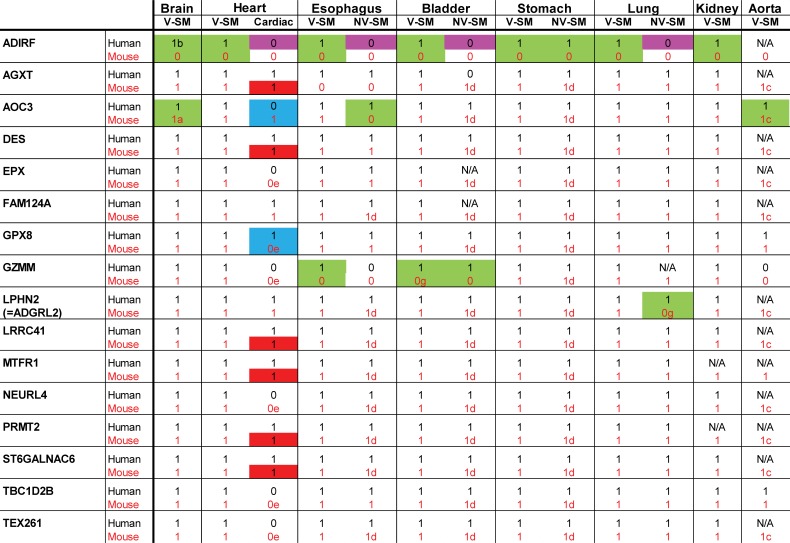
Summary of IHC staining of human and mouse tissues. We scored for the presence of significant and consistent staining using online data from the Human Protein Atlas for indicated human tissues. We scored mouse tissues stained for the same molecules in a blinded fashion. The presence of protein staining is indicated by 1, while the absence is indicated by 0. N/A indicates that staining was equivocal or inconsistent between samples or that the data was not available. Color codes and letters represent cases of non-conservation between cell types or species that are as follows: purple highlights show proteins expressed in VSMC but not in non-vascular SMC of the same organ in humans only; green highlights show proteins with clearcut differences in cellular staining between human and mouse tissues; blue highlights show proteins that exhibit differences in cardiomyoctes between human and mouse tissues; and red highlights show proteins that exhibited heterogeneity in cardiomyocytes. a: decreased in brain (vs. peripheral) VSMC, b: increased in brain (vs. peripheral) VSMC, c: heterogeneous in VSMC, d: heterogeneous in non-vascular SMC, e: weak, equivocal. V-SM indicates VSMC; NV-SM indicates non-vascular SMC.

Our original work led to the recognition that dozens of novel proteins in the brain were localized to VSMC or EC/VSMC [3]. We selected 18 of the most specific and robust antibodies that identified proteins in human VSMC of the brain; this set excluded canonical markers that have been extensively studied. These antibodies were then used to study protein distribution in mice. We observed staining in VSMC of multiple organs of mouse for 17 antibodies, demonstrating that IHC reagents were reactive against proteins from both human and mouse tissues. Western blot screens were performed to assess if the IHC patterns correlated with protein levels assessed by the same antibodies. Two of the original 18 proteins (HRC and RHOF) were excluded from the IHC analysis because expression intensities by IHC were not concordant with quantified protein levels when we examined bands on WB at the expected molecular masses (S7 and S8 Figs). The expression patterns in mouse tissues for the remaining 16 proteins that were suitable for comparison to human tissues are displayed in Figs 1 and 2. Comparisons were made between human and mouse tissues with attention to VSMC and non-vascular SMC. We scored for unambiguous differences between VSMC and non-vascular SMC within one organ, and we also looked for unambiguous evidence of SMC heterogeneity within one organ. The results are summarized in Fig 3.

### Relative VSMC versus non-vascular SMC protein expression in mice

Several proteins were shown before [3] in humans to have clearly increased expression in VSMC compared to non-vascular SMC. A majority of human proteins showed comparable vascular vs. non-vascular SMC expression. Comparisons were therefore made between mouse and human for the relative intensity of VSMC vs. non-vascular SMC staining in stomach, esophagus, and bladder, which afforded side-by-side comparisons of VSMC and non-vascular SMC in the same experiment. Species differences of note included ADIRF, which was not found in any mouse tissues (Fig 1, top row; Fig 3, highlighted with purple).

### Organ distribution of protein expression in SMC

All 16 expression markers were selected based on expression in human brain VSMC. All markers were expressed in VSMC of peripheral organs as well [3]. We determined if there were differences in organ expression in mouse that would potentially distinguish human/murine SMC outside of the brain. We found notable differences observed between human/murine cellular distribution for four proteins (AOC3, GZMM, LPHN2/ADGRL2, and ADIRF), highlighted in Fig 3 with green.

**Fig 4:**
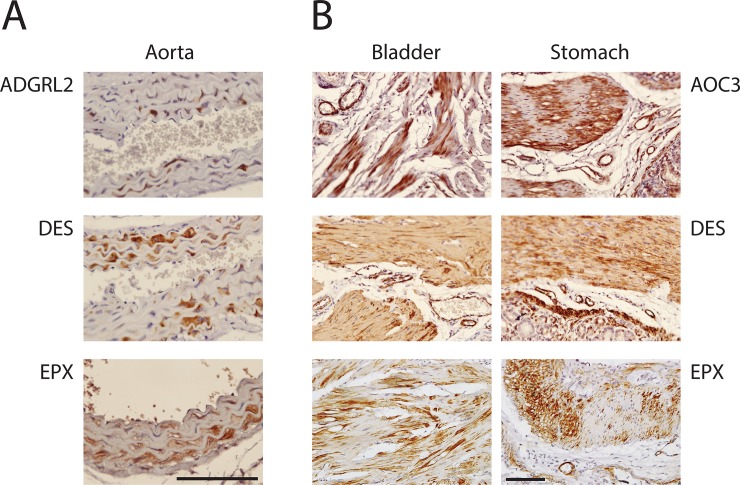
Smooth muscle cell heterogeneity in mouse. Several antibodies to smooth muscle proteins demonstrated heterogeneity of expression in VSMC that was clearly seen in the aorta (A) and in non-vascular SMC in bladder and stomach (B) of mouse.

First, AOC3 was expressed in all smooth muscle cells in all organs of the human. In mice, the expression pattern was partially restricted, with absence of convincing homogeneous staining in VSMC of the aorta and in non-vascular SMC of the esophagus. While brain AOC3 was present in some vessels of the mouse brain, it was noticeably decreased in cerebral VSMC compared to other vascular beds; in humans, AOC3 showed equal staining in brain and peripheral VSMC. In addition, staining of non-vascular SMC was heterogeneous (see stomach and bladder stains in Fig 4B, for example).

Second, GZMM was found in mouse brain, heart, kidney, stomach, and lung VSMC. However, it was largely absent in esophagus VSMC; this stands in contrast to human tissue, where GZMM was expressed in VSMC from all organs. In addition, in mouse, non-vascular SMC of the bladder and esophagus did not express GZMM; in humans, corresponding SMC of the bladder did express GZMM but esophagus did not. Finally, mouse did not stain for LPHN2/ADGRL2 in lung non-vascular SMC or for ADIRF in any tissue.

In aorta of mice, we observed strong staining for 14/16 markers (all were positive except GZMM and ADIRF); there was rare positivity of AOC3 (see Fig 1A). This finding identifies these AOC3 and GZMM as potential markers that can differentiate vascular smooth muscle of large vessels versus small vessels in mice. AOC3 (but not GZMM) was well-expressed in human aorta and is thus a molecule that is differentially expressed between human and mouse SMC in large vessels.

Cardiac muscle expresses a similar cohort of proteins as SMC. But the following proteins exhibited much stronger staining in VSMC of the mouse heart compared to adjacent cardiomyocytes: EPX, GPX8, GZMM, MTFR1, NEURL4, PRMT2, TBC1D2B, and TEX261. The same relationship was conserved in human heart tissue with the exception of GPX8, MTFR1, and PRMT2, which were expressed equally in cardiomyocytes and VSMC only in human. In contrast, AOC3 protein was expressed equally in cardiomyocytes and VSMC in mouse but in humans showed stronger staining in VSMC compared to adjacent cardiomyocytes. In total, two of 16 proteins showed species differences in cardiomyocytes (highlighted in blue in Fig 3). AGXT, DES, LRRC41, MTFR1, PRMT2, and ST6GALNAC6 protein was expressed heterogeneously in mouse cardiomyocytes, which was different from human (highlighted in red in Fig 3).

### Heterogeneity of SMC protein expression

Vascular SMC are known to exhibit at least two phenotypes [19], underlying vascular alterations in disease. Detailed analysis of staining patterns of the 16 proteins examined revealed widespread heterogeneity of expression in aorta for 11 proteins: AGXT, AOC3, DES, EPX, FAM124A, LPHN2/ADGRL2, LRRC41, NEURL4, PRMT2, ST6GALNAC6, and TEX261 showed clear evidence of high expressing cells adjacent to cells without detectable protein cells (Fig 4A).

Non-vascular smooth muscle cell heterogeneity was also observed in stomach and bladder for all markers that stained SMC. There was variation in the level of heterogeneity, with AOC3, DES, and EPX demonstrating remarkable variation between adjacent SMC cells (Fig 4B). Of note, three proteins showed heterogeneous expression in non-vascular SMC but not in SMC of aorta (GPX8, MTFR1, and TBC1D2B).

### Deletion of VSMC gene encoding sequences in mice

**Fig 5:**
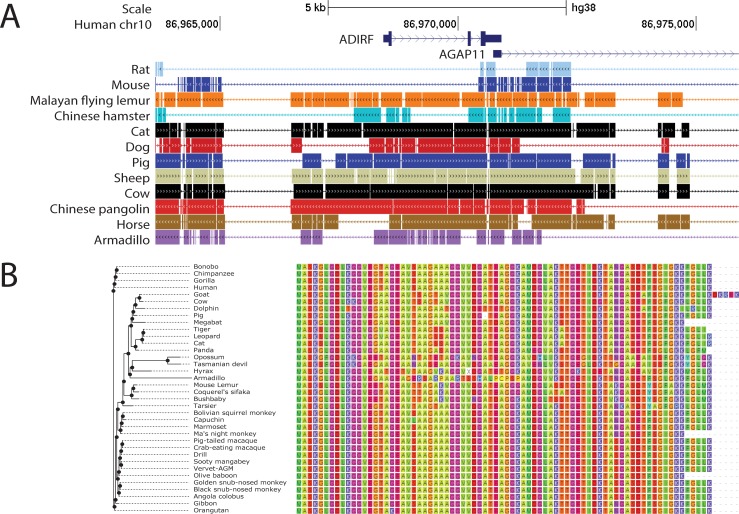
Cross-species molecular analysis of ADIRF. (A) Genomic ADIRF human locus is shown on top, indicating additional genes surrounding this segment of chromosome 10. Below, regions of DNA that are deleted in indicated species (including common experimental models, mouse and rat) are indicated by thin lines. Scale bar represents 5 kb. (B) Predicted amino acid sequences of ADIRF gene products in a variety of species is shown, as predicted by Ensembl. Amino acid classes are color coded to facilitate visual representation of cross-species conservation.

Inspection of publically available genome sequences reveals that the absence of ADIRF protein in mice arises through genetic differences between human and mouse. The mouse locus corresponding to human ADIRF harbors a deletion of close to 43 kb (Fig 5A). This deletion is predicted to remove the promoter and first exon of ADIRF, a sequence that encodes the first 42 amino acids of the 76 amino acid gene product. Of note, ADIRF is absent in several other vertebrate species (rat, zebrafish, lamprey) that also demonstrate deletions of this segment of DNA. However, among species where the gene is intact, the ADIRF predicted protein sequence appears to be very well conserved (Fig 5B). It is likely that the deletion of this segment (and loss of ADIRF expression) occurred in the evolutionary predecessor to mouse, rat, and hamster.

### Identification of 54 non-uniformly conserved human vascular proteins

We hypothesized that besides tissue level differences in expression patterns of vascular proteins, genomic differences could identify species differences in vascular molecules. To determine if other human cerebrovascular proteins, like ADIRF, were absent from non-human species, we focused on 346 brain vascular factors identified in a prior study (Tables 1–4 from [3]). A survey of three annotated genome databases was conducted for orthologues of each of these genes which revealed that 54 of the 346 protein coding genes (15.6%) do not have corresponding orthologues in at least one of 10 non-human vertebrate species queried. Tables 1–3 show proteins that were non-uniformly conserved by their location in human brain: EC only, VSMC/EC, VSMC only, respectively. ULBP2 was the only perivascular protein that was non-uniformly conserved. In total, 25 of these factors did not have orthologues in mouse annotated in Ensembl (see asterisks in Tables 1–3). Non-uniformly conserved VSMC proteins were much more likely to be absent in mouse than EC or VSMC/EC non-conserved molecules (84.6% for VSMC, compared with 19% for EC proteins and 42.1% for VSMC/EC proteins). The human VSMC/EC gene TIAF1 and EC gene ARHGEF35 did not have annotated orthologues in any of the 10 species queried. S1 Fig shows a full analysis of homology of orthologues from 10 species to respective human gene products.

**Table 1:** Overall differences between 10 vertebrate species and human with respect to the 21 non-uniformly conserved human proteins in EC only. (* denotes absence of mouse orthologue, 4 out of 21).

Gene	No Close Orthologue in
A2M	platypus, opossum
C12orf49	opossum, platypus
CABP7	opossum, platypus
CCDC71	cat, orangutan, opossum, platypus
CRB3*	mouse, rat, dog, cow, opossum, platypus
CRYBA2	opossum, platypus
FAM100B	dog, platypus
GIMAP1*	mouse, rat, pig, dog, cat, cow, chimpanzee, opossum, platypus
GRRP1	opossum, platypus
LONRF1	opossum
MYADMIL2	platypus
PODXL*	mouse, rat, pig, dog, cat, cow, opossum, platypus
RLN3	dog, opossum, platypus
S100A10	platypus
SCNM1	platypus
SDPR	platypus
TAPBP	opossum, platypus
TRIM59	chimpanzee, orangutan, opossum, platypus
TRIM73*	mouse, rat, pig, dog, cat, cow, opossum, platypus
VAMP5	orangutan, opossum, platypus
ZBTB5	platypus

**Table 2:** Overall differences between 10 vertebrate species and human with respect to the 19 non-uniformly conserved human proteins both in EC and SMC. (* denotes absence of mouse orthologue, 10 out of 19).

Gene	No Close Orthologue in
ALG1L*	mouse, rat, pig, dog, cat, cow, orangutan, opossum, platypus
AVPI1	platypus
C9orf47	platypus
C9orf75*	mouse, rat, pig, dog, cat, cow, opossum, platypus
C19orf60*	rat, dog, opossum, platypus
CCDC140*	mouse, rat, pig, dog, cat, cow, orangutan, opossum, platypus
GPER	chimpanzee, orangutan
JMJD4	cat, opossum, platypus
LDLRAD2*	mouse, rat, pig, opossum, platypus
LMTK3*	rat, cat, opossum
MAS1L*	mouse, rat, pig, dog, cat, cow, chimpanzee, opossum, platypus
NAB1	platypus
NES*	mouse, rat, pig, dog, cat, cow, opossum, platypus
NMB*	mouse, rat, dog, opossum, platypus
PNRC2	dog, cat, chimpanzee, platypus
SNAP47	mouse, cat, opossum, platypus
SYNPO2L	platypus
TIAF1*	mouse, rat, pig, dog, cat, cow, chimpanzee, orangutan, opossum, platypus
ZNF7	mouse, opossum, platypus

**Table 3:** Overall differences between 10 vertebrate species and human with respect to the 13 non-uniformly conserved human proteins in SMC only. (* denotes absence of mouse orthologue, 11 out of 13).

Gene	No Close Orthologue in
ARGFX*	mouse, rat, pig, dog, cat, cow, opossum, platypus
C9orf152*	mouse, rat, dog, cow, opossum, platypus
C17orf78*	mouse, rat, pig, cat, cow, opossum, platypus
CT47A11*	mouse, rat, pig, dog, cat, cow, opossum, platypus
DMRTC1B*	mouse, rat, pig, dog, cat, cow, opossum, platypus
HRC*	mouse, rat, dog, cat, cow, opossum, platypus
IFNA2*	mouse, rat, pig, dog, cat, cow, opossum, platypus
MAP7D3*	mouse, rat, pig, dog, cat, cow, opossum, platypus
RAPSN	pig, opossum
SPANXA1*	mouse, rat, pig, dog, cat, cow, orangutan, opossum, platypus
TSPYL1*	mouse, rat, pig, dog, opossum, platypus
ZC3HAV1L	cat, opossum, platypus
ZNF527*	mouse, rat, opossum, platypus

As noted above, human ADIRF lacks a murine orthologue because of a genomic deletion. Inspection of mouse genomic loci corresponding to the non-uniformly conserved vascular proteins demonstrated that additional deletions in the mouse genome, relative to the human genome, could account for the absence of gene products in mice. Murine genomic loci corresponding to three human smooth muscle-expressing loci harbored significant deletions predicted to disrupt protein coding sequences (ARGFX, SPANXA1, MA1L). The corresponding murine genomic sequence of one perivascular molecule, ULBP2, contained a deletion corresponding to the 5’ coding exon of the human sequence. In sum, 5/54 of the non-uniformly conserved vascular factors are disrupted in mouse due to large scale genomic deletions.

### Divergence of human and mouse VSMC protein sequences

**Fig 6:**
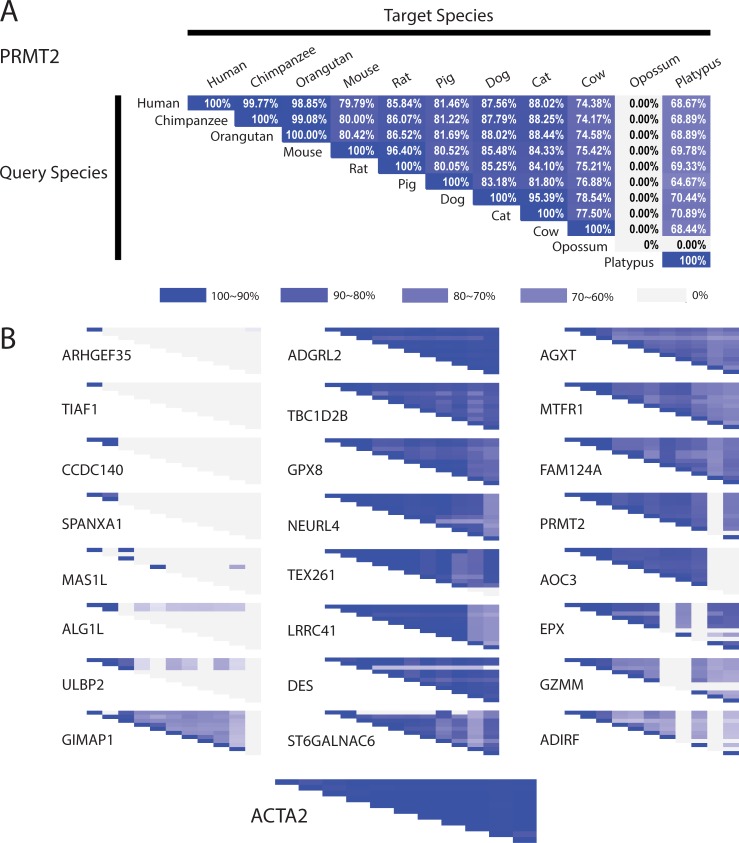
Cross-species analysis of SMC gene products. (A) Each matrix shows the degree of conservation of predicted SMC gene product of PRMT2 among different species; white/grey indicates no homology, whereas deep blue indicates complete homology (as shown on the diagonal). White boxes indicate the absence of an orthologue in the non-human species. (B) Matrices are shown for 25 SMC proteins from [3] that showed the lowest degrees of homology from 54 non-uniformly conserved human vascular proteins (left column) and 16 SMC proteins from Figs 1 and 2 (middle and right columns). On the bottom we show the highly conserved SMC protein, ACTA2 for comparison.

Many of the 54 non-uniformly conserved human vascular proteins noted above exhibited significant cross-species sequence variation (S1 Fig). We illustrate this by displaying the degree of protein sequence conservation of a sample of these proteins in pairwise comparisons of identity between 11 different species (10 above plus human) as annotated in Ensembl (Fig 6B, left column). We performed the same analysis of the 16 proteins analyzed in Figs 1 and 2 (Fig 6, middle and right columns). Cross-species similarity of all shown protein sequences demonstrates expected stronger conservation between humans and primates relative to rodents. The most conserved proteins exhibited high levels of similarity among all species. However, the most divergent proteins (relative to human/mouse conservation) exhibited very low conservation between human and all species except primates. The conservation of the 16 proteins examined above in Figs 1 and 2 was heterogeneous, with some proteins showing relatively low conservation (PRMT2, AOC3, EPX, GZMM, and ADIRF), even in experimental models such as mouse and rat. In contrast, the common SMC marker ACTA2 shows very strong conservation. Numerical values are shown in S2 Fig.

**Fig 7:**
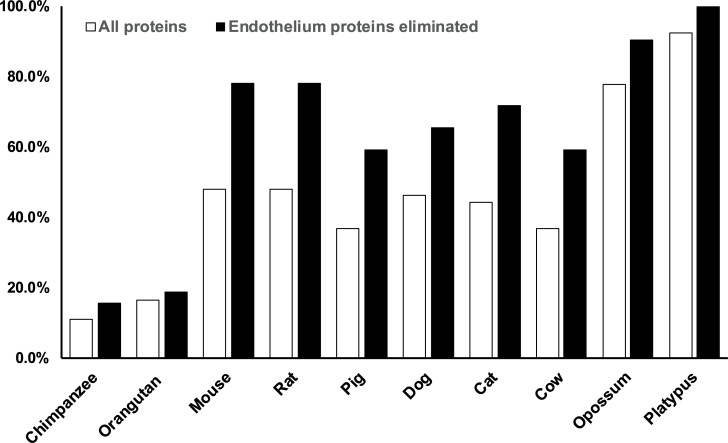
Divergence of a set of vascular proteins from human among 10 species. We analyzed the 54 proteins that demonstrated lack of an orthologue in at least one species. For each species, we summed the number of genes without an orthologue with the number of genes with sequence similarity (versus human) under 70%. The graph displays the percentage of proteins which are predicted to be absent or highly divergent for each species listed. White bars show divergence of all proteins in the set of 54; black bars show divergence of the combination of EC/VSMC and VSMC-only proteins (32 of the 54 proteins).

Finally, we assessed the overall differences between 10 vertebrate species and human with respect to the 54 non-uniformly conserved human vascular proteins. For each of the 10 species analyzed, we identified human genes with 1) no orthologues or 2) significant divergence of gene product sequence (<70% similarity compared to human) across 54 non-uniformly conserved human vascular genes (listed in Tables 1–3). When considering proteins in SMC (subset of 32 found in either VSMC only or VSMC/EC; Tables 2 and 3), 78% of these human proteins were either absent or divergent in mouse and rat (black bars in Fig 7). This level of divergence between mouse/rat and human, at this threshold, was slightly higher than what was seen for cat, dog, pig, and cow. Primates, chimpanzees and orangutans, were notably better conserved by this measure, and platypus and opossum had higher divergence (Fig 7; SMC analysis). When all proteins (including EC proteins) were analyzed in a similar way, mouse and rat differences decreased to 48% and was in the middle road among vertebrate species analyzed (white bars; Fig 7). All proteins absent or divergent from mouse or rat were found in VSMC, except a single protein, TRIM73, which was found only in EC. For detailed analysis, see S3 and S4 Figs, that display divergent genes (by species) and divergent species (by gene), respectively. This analysis shows that mouse and rat have high levels of divergence of vascular protein sequences among mammals and that proteins expressed in SMC exhibit substantially higher degrees of sequence divergence than EC proteins across all species.

An overview of the divergent (<70% similarity) proteins (relative to humans; S5 Fig) shows that mouse and rat share differences with humans, with only four exceptions (2 proteins were divergent in rats but not mice and 2 in mice but not rats). There were 15 primate specific proteins identified. There were 6 examples of proteins that diverged from human only in chimp not orangutan, while 3 proteins diverged only in orangutan but not chimp. Three proteins (GPER, TIAF1, and TRIM59) were absent in both species.

## Discussion

Conservation of murine and human biology is required in translational research. But the extent of similarity between the two species is not fully understood and likely varies depending on cell and tissue type. This study focuses on the level of species conservation of a series of 16 human SMC proteins using immunohistochemistry and sequence comparison. The main new findings include the identification of: 1) multiple differences between human and mouse SMC protein distributions on a tissue, region, and cell basis; 2) several proteins found to be expressed only in humans due to genetic deletions in mice; and 3) high primary sequence divergence between human and mouse in SMC proteins.

### Incomplete conservation of protein expression patterns

To maximize translational utility of murine studies, the expression pattern of SMC of humans and mice should ideally match. To test this, we used a set of antibodies that localize human SMC proteins on mouse tissues. Our results demonstrate that at the protein expression level, four out of 16 proteins lacked complete human/mouse SMC conservation, showing differences in SMC expression in an organ-dependent manner (ADIRF, AOC3, GZMM, and LPHN2/ADGRL2).

GZMM is found in human bladder SMC, but in mouse, it is absent from VSMC and non-vascular SMC of bladder. This protein is a protease that is implicated in cell killing when expressed in inflammatory cells [20]. But in smooth muscle it is more likely to regulate protein:protein interactions, as in plasma where it modulates binding of hemostatic proteins vWF and F8 [21]. The mouse/human differences of GZMM could decrease the translatability of studies of experimental murine models.

The differences we have noted at the protein level between human and mouse corresponding tissues may be a result of species difference in protein post-translational regulation (eg. degradation) or transcriptional regulation. Pishesha et al. has shown dramatic differences in transcriptional profiles of orthologous cells from mouse and humans [22], emphasizing the differences between the species even in fundamental process (in this case, erythropoiesis). More pertinent to this study, Lin et al performed tissue to tissue analysis between mouse and human RNA and found a much greater than expected difference between the two species [23]. Although this has been debated more recently based on technical considerations [24], a significant cohort of species transcript differences still remains even by conservative standards. These prior data suggest that many of the differences we observe in this study could be based on species transcriptional differences.

An additional layer of diversity could also arise in tissues due to cell-cell heterogeneous expression, found for multiple proteins in arteries of the mouse. Previous studies have noted a difference from cell to cell for several markers in the aorta [25] and in a vessel-dependent fashion in human fetuses and adult arteries for DES but not VIM [26, 27]; similar findings were noted in rats and rabbits for DES [28]. In addition, more recent studies using single cell analysis has clearly identified distinct populations of SMC by virtue of differential GPCR expression using PCR amplification and next-generation sequencing [29]. Our studies provide support at the protein level that SMC are not a homogenous population of cells, even in normal tissues, but vary widely within the vessel wall. These parallel findings that even within a confined location of the body (a vessel segment), attests to the potential vast diversity of this cell type that can drift between species. Our studies provide a panel of new tools that can be used to characterize protein-level SMC heterogeneity. It is expected that, based on the large proportion of the small number of markers studies herewith that were found to be heterogeneous, additional factors that vary between cells remain to be discovered.

### Species differences arising through genetic alterations

The most notable human/mouse SMC difference was the absence of ADIRF expression in mice. This protein was first identified in adipose tissue where it can regulate C/EBPa and PPARg, factors that affect smooth muscle homeostasis [30]. The ADIRF gene is relatively highly expressed (over 700 RKMP in tibial artery in GTEx [Release V6]) and more abundant in arterial than adipose tissue where it was initially characterized. Whether SMC in humans and mice differ in activity of the same regulatory pathways is a key question for future studies.

Genes that are present only in humans but not mice have been identified but are not common. Our studies indicated that several genes from SMC appear to be present in human but not mice and are worthy of detailed investigation. Several gene families differ between human and mouse, and these differences may not be important because of functional overlap between paralogs. The chances that ADIRF could result in functional changes is increased because it appears to encode a protein with no clear paralogs. One of the limitations of our analysis is that this approach does not identify proteins made in mice but not in humans. These factors may be significant, as early analysis showed that 14 of 731 mouse genes on Chr 16 have no human counterpart [31]. After the publication of both the human and mouse complete genomes, global estimates of protein coding genes unique to one species have increased sharply. Breschi et al reported that of the 19,950 protein coding human genes, 72% have one-to-one mouse homologues [13]. While some of the remaining genes have “many-to-many” relations (human and mice both have a many gene family, with mouse having lost some copies), there are likely many human proteins that do not have mouse orthologues besides ADIRF [32].

### Protein sequence differences in SMC molecules

The typical mouse protein, inferred from gene sequencing, is 70–95% identical to its human orthologue, with few exceptions. Among SMC proteins identified here, we show that a large fraction has less than 55% identity, demonstrating that this fraction of the proteome diverges more than would be expected by chance. Prior analysis showed that protein sequences are 85% identical between mouse and human ([33] (85.4+/-12.6 n = 1196); [34](range 41–100 mean 86.4+/-12.3% n = 2820)). The alteration in protein sequence may alter protein function in ways that differentiate human and mouse tissues. Formal determination of consequences of primary sequence require protein by protein substitution of genes in cells or in intact organisms.

### Future directions

These studies carry limitations. The principle one is that only a modest number of human markers were selected for analysis. We selected the strongest markers among the several hundred that were identified to assess the possibility of any differences and found, based on a modest number of proteins, that species divergence was easy to identify. Other studies should be performed on a) more proteins, b) more tissues/cell types, c) higher diversity of races/strains and ages, d) functional roles of the SMC proteins that demonstrate species difference, especially as they relate to SMC differentiation. It is anticipated, by extrapolation, that an expanded, broader study of 150 or more proteins may yield 30 human/mouse proteins with differences in SMC distribution. The two step approach we have taken, first identifying new proteins by visual inspection of the HPA and second using IHC screening to assess mouse/human expression, can be applied to other cell types to more broadly comprehend mouse/human protein differences. The species differences of 2 of 16 proteins in myocardium of heart, which was also scored in this study, is consistent with the need for independent work to be performed on other tissue systems.

In conclusion, we have uncovered molecular differences between mouse and human SMC using a modest panel of protein markers. Three dimensions of divergence between human and mouse have been highlighted by these markers. It is uncertain whether these expression result in altered phenotypes in mouse models. But the identification of these proteins offer potential explanations for failures of some mouse models in replicating disease or identifying human therapies. Human/mouse differences identified here can be overcome using genetic manipulation of mice to delete, add, or substitute human regulatory and/or structural sequences towards “humanization” of mice.

## Supporting information

S1 Fig Supplemental data 1. (PDF)

S2 Fig Supplemental data 2. (PDF)

S3 Fig Supplemental data 3. (PDF)

S4 Fig Supplemental data 4. (PDF)

S5 Fig Supplemental data 5. (PDF)

S6 Fig Supplemental data 6. (PDF)

S7 Fig Supplemental data 7. (PDF)

S8 Fig Supplemental data 8. (PDF)
