# Supplementary material for: Tripartite factors leading to molecular divergence between human and murine smooth muscle
Source: PLoS One. 2020 Jan 16;15(1):e0227672. doi: 10.1371/journal.pone.0227672 (PMC6964862; doi:10.1371/journal.pone.0227672)
Supplement: S1 Fig — (PDF) [file pone.0227672.s001.pdf]

Gene	mouse	rat	pig	dog	cat	cow	chimpanzee	orangutan	oppossum	platypus
A2M	71.98%	73.30%	76.26%	79.12%	79.74%	76.69%	99.12%	97.76%	59.83%	40.99%
ALG1L	26.97%	10.89%	29.06%	29.96%	30.39%	28.45%	94.71%	N/A	26.78%	N/A
ARGFX	N/A	N/A	61.60%	N/A	N/A	61.86%	97.46%	94.62%	N/A	N/A
AVPI1	76.76%	75%	90.54%	83.04%	87.16%	86.81%	97.28%	94.56%	72.54%	N/A
C9orf47	87.57%	87.34%	87%	82.54%	86.81%	87.30%	99.43%	98.94%	75.79%	N/A
C9orf75	57.01%	56.31%	54.12%	64.71%	N/A	63.82%	89.27%	93.32%	36.53%	N/A
C9orf152	62.29%	62.39%	74.48%	51.52%	73.64%	69.49%	98.33%	95.87%	N/A	N/A
C12orf49	90.24%	89.76%	94.15%	92.20%	93.17%	96.10%	99.51%	100%	66.99%	N/A
C17orf78	58.97%	62.07%	69.69%	72.92%	58.73%	66.43%	96.76%	94.96%	47.22%	N/A
C19orf60	70.41%	66.39%	74.09%	60.26%	80%	81.67%	98.01%	98.88%	N/A	N/A
CABP7	100%	100%	100%	100%	100%	100%	100%	100%	59.33%	N/A
CCDC71	74.83%	72.48%	81.28%	84.48%	N/A	81.42%	99.34%	N/A	56.15%	45.47%
CCDC140	N/A	N/A	N/A	N/A	N/A	N/A	98.77%	N/A	N/A	N/A
CRB3	64.10%	63.56%	76.86%	N/A	77.24%	N/A	77.60%	90.24%	N/A	N/A
CRYBA2	90.86%	91.88%	90.36%	78.51%	91.37%	90.86%	98.48%	95.94%	N/A	N/A
CT47A11	N/A	N/A	N/A	23.23%	N/A	27.01%	91.41%	89.35%	N/A	N/A
DMRTC1B	N/A	N/A	N/A	N/A	N/A	N/A	100%	100%	N/A	N/A
FAM100B	86.59%	87.73%	91.57%	N/A	93.37%	93.98%	100%	100%	88.48%	N/A
GIMAP1	61.67%	59.06%	60.20%	62.37%	59.86%	60.88%	N/A	83.24%	37.05%	N/A
GPER	86.40%	85.87%	79.17%	83.85%	87.36%	83.71%	N/A	N/A	77.39%	77.66%
GRRP1	100%	98.52%	92.80%	80.36%	82.85%	78.89%	87.08%	87.82%	N/A	N/A
HRC	50.14%	47.08%	70.48%	62.23%	N/A	55.11%	96.25%	79.53%	N/A	36.62%
IFNA2	60.85%	57.81%	56%	N/A	N/A	60.84%	98.24%	94.68%	N/A	N/A
JMJD4	74%	75.35%	74.76%	78.57%	69.98%	75.71%	98.70%	97.19%	57.11%	N/A
LDLRAD2	N/A	N/A	N/A	72.20%	77.39%	74.73%	97.43%	75.74%	59.28%	N/A
LMTK3	89.75%	N/A	93.09%	84.70%	N/A	93.35%	97.92%	99.30%	N/A	88.71%
LONRF1	80.88%	80.79%	91.30%	89.60%	92.22%	92.48%	99.61%	99.46%	N/A	82.93%
MAP7D3	27.63%	29.25%	29.06%	43.27%	48.92%	47.66%	97.37%	96.79%	28.01%	28.26%
MAS1L	N/A	N/A	25.81%	28.98%	N/A	29.11%	N/A	87.20%	26.65%	N/A
MYADML2	77.78%	92.51%	82.66%	91.21%	70.57%	90.88%	99.67%	97.69%	80.86%	N/A
NAB1	92.80%	92.78%	91.19%	85.22%	92.25%	95.89%	100%	99.79%	91.37%	N/A
NES	49.41%	47.91%	67.04%	66.77%	N/A	59.13%	98.70%	95.59%	37.10%	N/A
NMB	67.18%	67.52%	71.90%	65.06%	73.77%	72.73%	98.05%	94.16%	44.44%	N/A
PNRC2	77.14%	70.51%	95.68%	62.50%	N/A	93.53%	N/A	97.12%	80.85%	N/A
PODXL	42.15%	41.65%	45.15%	40.14%	41.12%	39.68%	95.14%	80.70%	30.98%	N/A
RAPSN	96.12%	96.60%	N/A	96.36%	97.09%	96.84%	99.76%	98.79%	N/A	83.21%
RLN3	75.89%	73.57%	78.57%	N/A	78.10%	72.06%	97.89%	94.37%	N/A	27.03%
S100A10	90.72%	89.47%	100%	83.33%	98.97%	100%	100%	100%	96.91%	N/A
SCNM1	81.18%	82.53%	88.70%	86.52%	88.72%	90.43%	99.13%	71.09%	70.69%	N/A
SDPR	84.21%	83.21%	87.50%	72.56%	89.18%	79.35%	99.53%	99.06%	73.13%	N/A
SNAP47	67.80%	73.27%	71.25%	75%	69.93%	77.86%	99.14%	96.30%	69.06%	N/A
SPANXA1	N/A	N/A	N/A	N/A	N/A	N/A	76.29%	N/A	N/A	N/A
SYNPO2L	87.83%	87.42%	90.98%	88.74%	92.09%	88.39%	99.59%	95.11%	78.96%	52.49%
TAPBP	71.24%	72.41%	73.52%	79.73%	81.51%	82.33%	94.42%	96.65%	55.56%	N/A
TIAF1	N/A	N/A	N/A	N/A	N/A	N/A	N/A	N/A	N/A	N/A
TRIM59	82.63%	83.87%	86.85%	88.12%	90.40%	89.75%	N/A	N/A	58.46%	60.75%
TRIM73	N/A	N/A	N/A	N/A	N/A	N/A	94%	87.20%	N/A	N/A
TSPYL1	67.02%	68.07%	66.59%	68.88%	70.40%	70.37%	98.63%	90.05%	N/A	N/A
ULBP2	14.93%	13.86%	32.54%	32.11%	N/A	38.89%	92.28%	N/A	32.95%	N/A
VAMP5	72.55%	70.30%	74.14%	75.65%	76.72%	75%	98.28%	41.90%	N/A	59.80%
ZBTB5	94.03%	93.88%	94.53%	97.05%	95.61%	95.41%	99.70%	99.70%	87.87%	N/A
ZC3HAV1L	75%	76.67%	79.67%	88.89%	69.25%	81.06%	99.33%	99%	48.72%	N/A
ZNF527	N/A	N/A	88.51%	89.75%	89.16%	84.23%	99.18%	98.69%	N/A	N/A
ZNF7	69.34%	79.73%	79.52%	82.67%	82.51%	78.63%	99%	97.52%	N/A	N/A

S1 Fig. Conservation of orthologues for 54 non-uniformaly conserved human vascular proteins. We analyzed 346 brain vascular factors and identified 54 that did not have orhologues in at least one of 10 non-human vertebrate species. The percentage identify between the predicted human protein sequence and non-human ortholgues are displayed. N/A is listed when no orthologue was present in the indicated species.
