# Supplementary material for: Tripartite factors leading to molecular divergence between human and murine smooth muscle
Source: PLoS One. 2020 Jan 16;15(1):e0227672. doi: 10.1371/journal.pone.0227672 (PMC6964862; doi:10.1371/journal.pone.0227672)
Supplement: S2 Fig — (PDF) [file pone.0227672.s002.pdf]

ARHGEF35	Human	Chimpanzee	Orangutan	Mouse	Rat	Pig	Dog	Cat	Cow	Opossum	Platypus
Human	100%	N/A	N/A	N/A	N/A	N/A	N/A	N/A	N/A	N/A	6.76%
Chimpanzee	N/A	N/A	N/A	N/A	N/A	N/A	N/A	N/A	N/A	N/A	N/A
Orangutan	N/A	N/A	N/A	N/A	N/A	N/A	N/A	N/A	N/A	N/A	N/A
Mouse	N/A	N/A	N/A	N/A	N/A	N/A	N/A	N/A	N/A	N/A	N/A
Rat	N/A	N/A	N/A	N/A	N/A	N/A	N/A	N/A	N/A	N/A	N/A
Pig	N/A	N/A	N/A	N/A	N/A	N/A	N/A	N/A	N/A	N/A	N/A
Dog	N/A	N/A	N/A	N/A	N/A	N/A	N/A	N/A	N/A	N/A	N/A
Cat	N/A	N/A	N/A	N/A	N/A	N/A	N/A	N/A	N/A	N/A	N/A
Cow	N/A	N/A	N/A	N/A	N/A	N/A	N/A	N/A	N/A	N/A	N/A
Opossum	N/A	N/A	N/A	N/A	N/A	N/A	N/A	N/A	N/A	N/A	N/A
Platypus	6.76%	N/A	N/A	N/A	N/A	N/A	N/A	N/A	N/A	N/A	N/A

TIAF1	Human	Chimpanzee	Orangutan	Mouse	Rat	Pig	Dog	Cat	Cow	Opossum	Platypus
Human	100%	N/A	N/A	N/A	N/A	N/A	N/A	N/A	N/A	N/A	N/A
Chimpanzee	N/A	N/A	N/A	N/A	N/A	N/A	N/A	N/A	N/A	N/A	N/A
Orangutan	N/A	N/A	N/A	N/A	N/A	N/A	N/A	N/A	N/A	N/A	N/A
Mouse	N/A	N/A	N/A	N/A	N/A	N/A	N/A	N/A	N/A	N/A	N/A
Rat	N/A	N/A	N/A	N/A	N/A	N/A	N/A	N/A	N/A	N/A	N/A
Pig	N/A	N/A	N/A	N/A	N/A	N/A	N/A	N/A	N/A	N/A	N/A
Dog	N/A	N/A	N/A	N/A	N/A	N/A	N/A	N/A	N/A	N/A	N/A
Cat	N/A	N/A	N/A	N/A	N/A	N/A	N/A	N/A	N/A	N/A	N/A
Cow	N/A	N/A	N/A	N/A	N/A	N/A	N/A	N/A	N/A	N/A	N/A
Opossum	N/A	N/A	N/A	N/A	N/A	N/A	N/A	N/A	N/A	N/A	N/A
Platypus	6.76%	N/A	N/A	N/A	N/A	N/A	N/A	N/A	N/A	N/A	N/A

CCDC140	Human	Chimpanzee	Orangutan	Mouse	Rat	Pig	Dog	Cat	Cow	Opossum	Platypus
Human	100%	98.77%	N/A	N/A	N/A	N/A	N/A	N/A	N/A	N/A	N/A
Chimpanzee	98.77%	100%	N/A	N/A	N/A	N/A	N/A	N/A	N/A	N/A	N/A
Orangutan	N/A	N/A	N/A	N/A	N/A	N/A	N/A	N/A	N/A	N/A	N/A
Mouse	N/A	N/A	N/A	N/A	N/A	N/A	N/A	N/A	N/A	N/A	N/A
Rat	N/A	N/A	N/A	N/A	N/A	N/A	N/A	N/A	N/A	N/A	N/A
Pig	N/A	N/A	N/A	N/A	N/A	N/A	N/A	N/A	N/A	N/A	N/A
Dog	N/A	N/A	N/A	N/A	N/A	N/A	N/A	N/A	N/A	N/A	N/A
Cat	N/A	N/A	N/A	N/A	N/A	N/A	N/A	N/A	N/A	N/A	N/A
Cow	N/A	N/A	N/A	N/A	N/A	N/A	N/A	N/A	N/A	N/A	N/A
Opossum	N/A	N/A	N/A	N/A	N/A	N/A	N/A	N/A	N/A	N/A	N/A
Platypus	6.76%	N/A	N/A	N/A	N/A	N/A	N/A	N/A	N/A	N/A	N/A

SPANXA1	Human	Chimpanzee	Orangutan	Mouse	Rat	Pig	Dog	Cat	Cow	Opossum	Platypus
Human	100%	76.29%	N/A	N/A	N/A	N/A	N/A	N/A	N/A	N/A	N/A
Chimpanzee	76.29%	100%	N/A	N/A	N/A	N/A	N/A	N/A	N/A	N/A	N/A
Orangutan	N/A	N/A	N/A	N/A	N/A	N/A	N/A	N/A	N/A	N/A	N/A
Mouse	N/A	N/A	N/A	N/A	N/A	N/A	N/A	N/A	N/A	N/A	N/A
Rat	N/A	N/A	N/A	N/A	N/A	N/A	N/A	N/A	N/A	N/A	N/A
Pig	N/A	N/A	N/A	N/A	N/A	N/A	N/A	N/A	N/A	N/A	N/A
Dog	N/A	N/A	N/A	N/A	N/A	N/A	N/A	N/A	N/A	N/A	N/A
Cat	N/A	N/A	N/A	N/A	N/A	N/A	N/A	N/A	N/A	N/A	N/A
Cow	N/A	N/A	N/A	N/A	N/A	N/A	N/A	N/A	N/A	N/A	N/A
Opossum	N/A	N/A	N/A	N/A	N/A	N/A	N/A	N/A	N/A	N/A	N/A
Platypus	6.76%	N/A	N/A	N/A	N/A	N/A	N/A	N/A	N/A	N/A	N/A

MAS1L	Human	Chimpanzee	Orangutan	Mouse	Rat	Pig	Dog	Cat	Cow	Opossum	Platypus
Human	100%	N/A	93.87%	N/A	N/A	N/A	N/A	N/A	N/A	N/A	N/A
Chimpanzee	N/A	N/A	N/A	N/A	N/A	N/A	N/A	N/A	N/A	N/A	N/A
Orangutan	93.87%	N/A	100%	N/A	N/A	N/A	N/A	N/A	N/A	N/A	N/A
Mouse	N/A	N/A	N/A	N/A	N/A	N/A	N/A	N/A	N/A	N/A	N/A
Rat	N/A	N/A	N/A	N/A	100%	N/A	N/A	N/A	N/A	N/A	N/A
Pig	N/A	N/A	N/A	N/A	N/A	N/A	N/A	N/A	N/A	N/A	N/A
Dog	N/A	N/A	N/A	N/A	N/A	N/A	N/A	N/A	N/A	N/A	N/A
Cat	N/A	N/A	N/A	N/A	N/A	N/A	N/A	N/A	N/A	N/A	N/A
Cow	N/A	N/A	N/A	N/A	N/A	N/A	N/A	N/A	N/A	N/A	N/A
Opossum	N/A	N/A	N/A	N/A	45.95%	N/A	N/A	N/A	N/A	N/A	N/A
Platypus	6.76%	N/A	N/A	N/A	N/A	N/A	N/A	N/A	N/A	N/A	N/A

ALG1L	Human	Chimpanzee	Orangutan	Mouse	Rat	Pig	Dog	Cat	Cow	Opossum	Platypus
Human	100%	92.28%	N/A	26.97%	10.89%	29.06%	29.96%	30.39%	28.45%	26.78%	N/A
Chimpanzee	92.28%	100%	N/A	27.80%	11.88%	29.49%	30.39%	30.82%	28.88%	27.86%	N/A
Orangutan	N/A	N/A	N/A	N/A	N/A	N/A	N/A	N/A	N/A	N/A	N/A
Mouse	26.97%	27.80%	N/A	N/A	N/A	N/A	N/A	N/A	N/A	N/A	N/A
Rat	10.89%	11.88%	N/A	N/A	N/A	N/A	N/A	N/A	N/A	N/A	N/A
Pig	29.06%	29.49%	N/A	N/A	N/A	N/A	N/A	N/A	N/A	N/A	N/A
Dog	29.96%	30.39%	N/A	N/A	N/A	N/A	N/A	N/A	N/A	N/A	N/A
Cat	30.39%	30.82%	N/A	N/A	N/A	N/A	N/A	N/A	N/A	N/A	N/A
Cow	28.45%	28.88%	N/A	N/A	N/A	N/A	N/A	N/A	N/A	N/A	N/A
Opossum	26.78%	27.86%	N/A	N/A	N/A	N/A	N/A	N/A	N/A	N/A	N/A
Platypus	N/A	N/A	N/A	N/A	N/A	N/A	N/A	N/A	N/A	N/A	N/A

ULBP2	Human	Chimpanzee	Orangutan	Mouse	Rat	Pig	Dog	Cat	Cow	Opossum	Platypus
Human	100%	92.28%	77.38%	16.47%	N/A	42.21%	28.28%	N/A	44.26%	17.87%	N/A
Chimpanzee	92.28%	100%	77.38%	16.77%	N/A	42.62%	28.28%	N/A	44.26%	16.43%	N/A
Orangutan	77.38%	77.38%	100%	16.77%	N/A	43.60%	31.75%	N/A	45.02%	18.36%	N/A
Mouse	16.47%	16.77%	16.77%	N/A	N/A	N/A	N/A	N/A	N/A	N/A	N/A
Rat	N/A	N/A	N/A	N/A	N/A	N/A	N/A	N/A	N/A	N/A	N/A
Pig	42.21%	42.62%	43.60%	N/A	N/A	N/A	N/A	N/A	N/A	N/A	N/A
Dog	28.28%	28.28%	31.75%	N/A	N/A	N/A	N/A	N/A	N/A	N/A	N/A
Cat	N/A	N/A	N/A	N/A	N/A	N/A	N/A	N/A	N/A	N/A	N/A
Cow	44.26%	44.26%	45.02%	N/A	N/A	N/A	N/A	N/A	N/A	N/A	N/A
Opossum	17.87%	16.43%	18.36%	N/A	N/A	N/A	N/A	N/A	N/A	N/A	N/A
Platypus	N/A	N/A	N/A	N/A	N/A	N/A	N/A	N/A	N/A	N/A	N/A

GIMAP1	Human	Chimpanzee	Orangutan	Mouse	Rat	Pig	Dog	Cat	Cow	Opossum	Platypus
Human	100%	N/A	83.24%	61.33%	59.06%	59.87%	61.69%	59.18%	57.74%	37.05%	N/A
Chimpanzee	N/A	100%	92.12%	55.48%	46.63%	54.83%	63.78%	58.58%	54.83%	45.60%	N/A
Orangutan	83.24%	92.12%	100.00%	61.00%	58.72%	59.53%	60%	58.16%	58.39%	39.38%	N/A
Mouse	61.33%	55.48%	61.00%	100%	79.87%	56.19%	57.97%	53.40%	54.19%	37.05%	N/A
Rat	59.06%	46.63%	58.72%	79.87%	100%	53.18%	54.24%	52.38%	53.06%	35.49%	N/A
Pig	59.87%	54.83%	59.53%	56.19%	53.18%	100%	56.95%	55.44%	61.94%	34.97%	N/A
Dog	61.69%	63.78%	60%	57.97%	54.24%	56.95%	100%	72.11%	58.06%	32.64%	N/A
Cat	59.18%	58.58%	58.16%	53.40%	52.38%	55.44%	72.11%	100%	58.71%	31.09%	N/A
Cow	57.74%	54.83%	58.39%	54.19%	53.06%	61.94%	58.06%	58.71%	100%	33.68%	N/A
Opossum	37.05%	45.60%	39.38%	37.05%	35.49%	34.97%	32.64%	31.09%	33.68%	100%	N/A
Platypus	N/A	N/A	N/A	N/A	N/A	N/A	N/A	N/A	N/A	N/A	N/A

ACTA2	Human	Chimpanzee	Orangutan	Mouse	Rat	Pig	Dog	Cat	Cow	Opossum	Platypus
Human	100%	86.68%	94.49%	100%	99.47%	100%	100%	100%	100%	95.15%	99.73%
Chimpanzee	86.68%	100.00%	89.72%	94.96%	94.41%	94.96%	94.96%	94.96%	94.96%	95.15%	94.69%
Orangutan	94.49%	89.72%	100%	94.49%	99.47%	100%	100%	100%	100%	95.15%	99.73%
Mouse	100%	94.96%	94.49%	100%	99.47%	100%	100%	100%	100%	95.15%	99.73%
Rat	99.47%	94.41%	99.47%	99.47%	100%	99.20%	99.20%	99.20%	99.20%	94.24%	98.94%
Pig	100%	94.96%	100%	100%	99.20%	100%	100%	100%	100%	95.15%	99.73%
Dog	100%	94.96%	100%	100%	99.20%	100%	100%	100%	100%	95.15%	99.73%
Cat	100%	94.96%	100%	100%	99.20%	100%	100%	100%	100%	95.15%	99.73%
Cow	100%	94.96%	100%	100%	99.20%	100%	100%	100%	100%	95.15%	99.73%
Opossum	95.15%	95.15%	95.15%	95.15%	94.24%	95.15%	95.15%	95.15%	95.15%	100%	83.02%
Platypus	99.73%	94.69%	99.73%	99.73%	98.94%	99.73%	99.73%	99.73%	99.73%	83.02%	100%

RHOF	Human	Chimpanzee	Orangutan	Mouse	Rat	Pig	Dog	Cat	Cow	Opossum	Platypus
Human	100%	99.05%	98.57%	92.89%	93.36%	95.26%	95.17%	94.20%	93.95%	90.71%	87.68%
Chimpanzee	99.05%	100%	98.57%	92.89%	93.36%	95.26%	95.17%	94.20%	93.95%	90.16%	87.68%
Orangutan	98.57%	98.57%	100%	93.36%	93.84%	95.73%	96.14%	95.17%	94.88%	89.62%	86.96%
Mouse	92.89%	92.89%	93.36%	100%	98.10%	91.94%	91.79%	91.79%	92.09%	88.52%	86.96%
Rat	93.36%	93.36%	93.84%	98.10%	100%	91.47%	91.30%	90.82%	92.09%	90.16%	87.68%
Pig	95.26%	95.26%	95.73%	91.94%	91.47%	100%	95.17%	96.14%	93.02%	89.07%	86.96%
Dog	95.17%	95.17%	96.14%	91.79%	91.30%	95.17%	100%	96.14%	92.56%	88.52%	84.78%
Cat	94.20%	94.20%	95.17%	91.79%	90.82%	96.14%	96.14%	100%	91.63%	87.98%	87.68%
Cow	93.95%	93.95%	94.88%	92.09%	92.09%	93.02%	92.56%	91.63%	100%	89.62%	86.96%
Opossum	90.71%	90.16%	89.62%	88.52%	90.16%	89.07%	88.52%	87.98%	89.62%	100%	86.23%
Platypus	87.68%	87.68%	86.96%	86.96%	87.68%	86.96%	84.78%	87.68%	86.96%	86.23%	100%

LPHN2(ADGRL2)	Human	Chimpanzee	Orangutan	Mouse	Rat	Pig	Dog	Cat	Cow	Opossum	Platypus
Human	100%	95.34%	95.49%	91.19%	90.17%	94.12%	98.23%	93.48%	93.93%	90.05%	90.35%
Chimpanzee	95.34%	100%	98.93%	95.09%	94.15%	98.04%	98.16%	97.38%	97.91%	94.01%	93.76%
Orangutan	95.49%	98.93%	100%	78.35%	77.32%	80.80%	81.50%	80.17%	80.38%	77.47%	93.35%
Mouse	91.19%	95.09%	78.35%	100%	96.70%	95.74%	95.76%	95.77%	95.68%	92.94%	92.70%
Rat	90.17%	94.15%	77.32%	96.70%	100%	94.52%	94.63%	94.42%	94.81%	91.86%	91.16%
Pig	94.12%	98.04%	80.80%	95.74%	94.52%	100%	99.01%	98.52%	98.58%	94.35%	94.65%
Dog	98.23%	98.16%	81.50%	95.76%	94.63%	99.01%	100%	94.15%	94.20%	90.18%	91.08%
Cat	93.48%	97.38%	80.17%	95.77%	94.42%	98.52%	94.15%	100%	98.58%	95.16%	94.48%
Cow	93.93%	97.91%	80.38%	95.68%	94.81%	98.58%	94.20%	98.58%	100%	94.55%	93.84%
Opossum	90.05%	94.01%	77.47%	92.94%	91.86%	94.35%	90.18%	95.16%	94.55%	100%	95.78%
Platypus	90.35%	93.76%	93.35%	92.70%	91.16%	94.65%	91.08%	94.48%	93.84%	95.78%	100%

TBC1D2B	Human	Chimpanzee	Orangutan	Mouse	Rat	Pig	Dog	Cat	Cow	Opossum	Platypus
Human	100%	99.90%	96.84%	88.60%	90.44%	89.92%	88.49%	87.14%	89.26%	81.39%	84.62%
Chimpanzee	99.90%	100%	96.84%	88.70%	90.44%	90.03%	88.49%	87.14%	89.26%	81.39%	84.62%
Orangutan	96.84%	96.84%	100%	70.98%	82.80%	81.23%	80.58%	70.56%	81.62%	66.70%	83.65%
Mouse	88.60%	88.70%	70.98%	100%	95.70%	86.44%	85.37%	84.27%	86.04%	78.70%	80.63%
Rat	90.44%	90.44%	82.80%	95.70%	100%	85.75%	86.57%	75.35%	86.28%	70.22%	81.04%
Pig	89.92%	90.03%	81.23%	86.44%	85.75%	100%	88.13%	78.96%	92.24%	74.66%	85.99%
Dog	88.49%	88.49%	80.58%	85.37%	86.57%	88.13%	100%	79.70%	86.04%	70.22%	82.01%
Cat	87.14%	87.14%	70.56%	84.27%	75.35%	78.96%	79.70%	100%	86.52%	78.18%	81.46%
Cow	89.26%	89.26%	81.62%	86.04%	86.28%	92.24%	86.04%	86.52%	100%	71.87%	83.24%
Opossum	81.39%	81.39%	66.70%	78.70%	70.22%	74.66%	70.22%	78.18%	71.87%	100%	87.09%
Platypus	84.62%	84.62%	83.65%	80.63%	81.04%	85.99%	82.01%	81.46%	83.24%	87.09%	100%

GPX8	Human	Chimpanzee	Orangutan	Mouse	Rat	Pig	Dog	Cat	Cow	Opossum	Platypus
Human	100%	99.04%	98.38%	82.30%	83.25%	90.91%	88.65%	91.39%	89%	78.57%	74.76%
Chimpanzee	99.04%	100%	99.46%	83.25%	84.21%	91.87%	86.60%	92.34%	89.95%	79.52%	75.24%
Orangutan	98.38%	99.46%	100%	73.21%	74.16%	82.30%	77.99%	82.78%	80.86%	71.43%	69.05%
Mouse	82.30%	83.25%	73.21%	100%	96.65%	83.25%	78.95%	83.25%	81.82%	75.71%	69.05%
Rat	83.25%	84.21%	74.16%	96.65%	100%	84.21%	79.90%	84.21%	83.25%	75.24%	69.52%
Pig	90.91%	91.87%	82.30%	83.25%	84.21%	100%	94.74%	98.56%	96.65%	77.62%	74.76%
Dog	88.65%	86.60%	77.99%	78.95%	79.90%	94.74%	100%	95.22%	93.78%	74.76%	74.76%
Cat	91.39%	92.34%	82.78%	83.25%	84.21%	98.56%	95.22%	100%	97.61%	77.14%	74.76%
Cow	89%	89.95%	80.86%	81.82%	83.25%	96.65%	93.78%	97.61%	100%	75.71%	72.86%
Opossum	78.57%	79.52%	71.43%	75.71%	75.24%	77.62%	74.76%	77.14%	75.71%	100%	76.67%
Platypus	74.76%	75.24%	69.05%	69.05%	69.52%	74.76%	74.76%	74.76%	72.86%	76.67%	100%

NEURL4	Human	Chimpanzee	Orangutan	Mouse	Rat	Pig	Dog	Cat	Cow	Opossum	Platypus
Human	100%	99.68%	99.30%	95.65%	95.64%	92.15%	95.12%	96.76%	88.51%	87.04%	60%
Chimpanzee	99.68%	100%	99.04%	95.39%	95.39%	91.93%	94.90%	96.50%	88.33%	86.90%	59.47%
Orangutan	99.30%	99.04%	100%	95.59%	95.58%	91.93%	94.90%	96.70%	88.39%	87.17%	60%
Mouse	95.65%	95.39%	95.59%	100%	98.53%	90.71%	93.21%	95.28%	87.32%	86.05%	60%
Rat	95.64%	95.39%	95.58%	98.53%	100%	90.71%	93.31%	95.15%	87.20%	85.65%	60%
Pig	92.15%	91.93%	91.93%	90.71%	90.71%	100%	94.59%	81.81%	75.89%	74.21%	62.63%
Dog	95.12%	94.90%	94.90%	93.21%	93.31%	94.59%	100%	57.28%	53.33%	52.78%	57.89%
Cat	96.76%	96.50%	96.70%	95.28%	95.15%	81.81%	57.28%	100%	87.44%	85.98%	52.63%
Cow	88.51%	88.33%	88.39%	87.32%	87.20%	75.89%	53.33%	87.44%	100%	85.98%	61.05%
Opossum	87.04%	86.90%	87.17%	86.05%	85.65%	74.21%	52.78%	85.98%	85.98%	100%	62.11%
Platypus	60%	59.47%	60%	60%	60%	62.63%	57.89%	52.63%	61.05%	62.11%	100%

TEX261	Human	Chimpanzee	Orangutan	Mouse	Rat	Pig	Dog	Cat	Cow	Opossum	Platypus
Human	100%	100%	100%	99.49%	99.49%	98.98%	89.29%	97.96%	98.47%	75.69%	93.62%
Chimpanzee	100%	100%	100%	99.49%	99.49%	98.98%	89.29%	98.47%	72.27%	75.69%	93.62%
Orangutan	100%	100%	100%	99.49%	99.49%	98.98%	89.29%	98.47%	72.27%	75.69%	93.62%
Mouse	99.49%	99.49%	99.49%	100%	100%	99.49%	89.29%	98.98%	72.69%	76.39%	93.62%
Rat	99.49%	99.49%	99.49%	100%	100%	99.49%	89.29%	98.98%	72.69%	76.39%	93.62%
Pig	98.98%	98.98%	98.98%	99.49%	99.49%	100%	88.78%	98.47%	72.27%	75.69%	93.62%
Dog	89.29%	89.29%	89.29%	89.29%	89.29%	88.78%	100%	89.29%	64.29%	65.97%	92.55%
Cat	97.96%	98.47%	98.47%	98.98%	98.98%	98.47%	89.29%	100%	72.27%	77.08%	92.55%
Cow	98.47%	72.27%	72.27%	72.69%	72.69%	72.27%	64.29%	72.27%	100%	76.39%	73.40%
Opossum	75.69%	75.69%	75.69%	76.39%	76.39%	75.69%	65.97%	77.08%	76.39%	N/A	N/A
Platypus	93.62%	93.62%	93.62%	93.62%	93.62%	93.62%	92.55%	92.55%	73.40%	N/A	N/A

LRRC41	Human	Chimpanzee	Orangutan	Mouse	Rat	Pig	Dog	Cat	Cow	Opossum	Platypus
Human	100%	100%	99.46%	95.42%	95.79%	96.89%	97.79%	98.28%	97.91%	60.49%	54.56%
Chimpanzee	100%	100%	99.46%	95.42%	95.79%	96.89%	97.79%	98.28%	97.91%	60.49%	54.56%
Orangutan	99.46%	99.46%	100%	85.87%	86.39%	87.20%	88.21%	88.70%	88.31%	53.84%	47.44%
Mouse	95.42%	95.42%	85.87%	100%	97.77%	94.53%	94.96%	94.96%	94.34%	59.97%	54.56%
Rat	95.79%	95.79%	86.39%	97.77%	100%	94.78%	95.58%	95.33%	94.96%	59.97%	54.43%
Pig	96.89%	96.89%	87.20%	94.53%	94.78%	100%	95.95%	96.56%	96.43%	60.74%	54.43%
Dog	97.79%	97.79%	88.21%	94.96%	95.58%	95.95%	100%	99.26%	98.16%	60.61%	54.68%
Cat	98.28%	98.28%	88.70%	94.96%	95.33%	96.56%	99.26%	100%	98.65%	60.61%	54.43%
Cow	97.91%	97.91%	88.31%	94.34%	94.96%	96.43%	98.16%	98.65%	100%	60.61%	54.81%
Opossum	60.49%	60.49%	53.84%	59.97%	59.97%	60.74%	60.61%	60.61%	60.61%	100%	47.19%
Platypus	54.56%	54.56%	47.44%	54.56%	54.43%	54.43%	54.68%	54.43%	54.81%	47.19%	100%

DES	Human	Chimpanzee	Orangutan	Mouse	Rat	Pig	Dog	Cat	Cow	Opossum	Platypus
Human	100%	88.22%	100%	97.65%	98.29%	81.32%	97.87%	88.91%	98.51%	94.07%	89.32%
Chimpanzee	88.22%	100%	98.69%	94.88%	95.52%	78.56%	95.10%	86.38%	95.74%	91.31%	89.32%
Orangutan	100%	98.69%	100%	32.41%	32.62%	32.48%	32.41%	29.77%	32.55%	31.99%	N/A
Mouse	97.65%	94.88%	32.41%	100%	99.36%	80.04%	96.38%	87.55%	97.02%	93.64%	89.32%
Rat	98.29%	95.52%	32.62%	99.36%	100%	80.25%	97.01%	88.13%	97.66%	93.43%	89.32%
Pig	81.32%	78.56%	32.48%	80.04%	80.25%	100%	81.24%	74.32%	81.70%	78.81%	76.70%
Dog	97.87%	95.10%	32.41%	96.38%	97.01%	81.24%	100%	88.13%	97.66%	92.80%	89.32%
Cat	88.91%	86.38%	29.77%	87.55%	88.13%	74.32%	88.13%	100%	97.45%	92.58%	90.29%
Cow	98.51%	95.74%	32.55%	97.02%	97.66%	81.70%	97.66%	97.45%	100%	93.36%	90.29%
Opossum	94.07%	91.31%	31.99%	93.64%	93.43%	78.81%	92.80%	92.58%	93.36%	100%	86.41%
Platypus	89.32%	89.32%	no orthologue	89.32%	89.32%	76.70%	89.32%	90.29%	90.29%	86.41%	100%

ST6GALNAC6	Human	Chimpanzee	Orangutan	Mouse	Rat	Pig	Dog	Cat	Cow	Opossum	Platypus
Human	100%	99.20%	N/A	N/A	N/A	N/A	N/A	87.40%	N/A	42.83%	65.71%
Chimpanzee	99.20%	100%	82.58%	75.38%	76.59%	77.58%	75.07%	86.85%	65.54%	43.24%	65.71%
Orangutan	N/A	82.58%	100%	90.09%	92.64%	92.42%	89.57%	69.59%	77.72%	51.14%	81.79%
Mouse	N/A	75.38%	90.09%	100%	97.32%	90.30%	89.28%	68.22%	77.20%	51.14%	81.07%
Rat	N/A	76.59%	92.64%	97.32%	100%	82.73%	82.90%	61.92%	71.50%	50.31%	80.71%
Pig	N/A	77.58%	92.42%	90.30%	82.73%	100%	88.41%	68.22%	78.50%	49.69%	78.21%
Dog	N/A	75.07%	89.57%	89.28%	82.90%	88.41%	100%	70.68%	78.24%	52.18%	82.14%
Cat	87.40%	86.85%	69.59%	68.22%	61.92%	68.22%	70.68%	100%	63.99%	41.79%	63.93%
Cow	N/A	65.54%	77.72%	77.20%	71.50%	78.50%	78.24%	63.99%	100%	50.73%	79.29%
Opossum	42.83%	43.24%	51.14%	51.14%	50.31%	49.69%	52.18%	41.79%	50.73%	100%	85.00%
Platypus	65.71%	65.71%	81.79%	81.07%	80.71%	78.21%	82.14%	63.93%	79.29%	85.00%	100%

AGXT	Human	Chimpanzee	Orangutan	Mouse	Rat	Pig	Dog	Cat	Cow	Opossum	Platypus
Human	100%	98.37%	84.31%	72.71%	75.12%	78.86%	75.85%	78.99%	76.57%	69.60%	73.24%
Chimpanzee	98.37%	100%	84.87%	67.87%	70.29%	73.63%	71.22%	74.40%	71.74%	64.13%	67.61%
Orangutan	84.31%	84.87%	100%	62.56%	64.25%	64.68%	64.15%	66.18%	63.53%	52.28%	54.93%
Mouse	72.71%	67.87%	62.56%	100%	93%	74.38%	72.93%	75.36%	72.46%	65.05%	66.76%
Rat	75.12%	70.29%	64.25%	93%	100%	77.11%	74.63%	77.05%	73.91%	65.96%	69.01%
Pig	78.86%	73.63%	64.68%	74.38%	77.11%	100%	78.05%	82.37%	79.71%	66.26%	71.55%
Dog	75.85%	71.22%	64.15%	72.93%	74.63%	78.05%	100%	82.37%	74.15%	65.05%	71.55%
Cat	78.99%	74.40%	66.18%	75.36%	77.05%	82.37%	82.37%	100%	79.95%	69%	76.06%
Cow	76.57%	71.74%	63.53%	72.46%	73.91%	79.71%	74.15%	79.95%	100%	68.39%	71.27%
Opossum	69.60%	64.13%	52.28%	65.05%	65.96%	66.26%	65.05%	69%	68.39%	100%	61.97%
Platypus	73.24%	67.61%	54.93%	66.76%	69.01%	71.55%	71.55%	76.06%	71.27%	61.97%	100%

MTFR1	Human	Chimpanzee	Orangutan	Mouse	Rat	Pig	Dog	Cat	Cow	Opossum	Platypus
Human	100%	98.80%	97.60%	77.44%	77.01%	72.91%	83.53%	85.67%	68.60%	66.13%	61.94%
Chimpanzee	98.80%	100%	98.20%	77.44%	77.31%	72.91%	83.83%	85.67%	68.31%	66.13%	62.22%
Orangutan	97.60%	98.20%	100%	76.83%	77.01%	73.49%	83.83%	85.37%	68.31%	66.77%	62.22%
Mouse	77.44%	77.44%	76.83%	100%	87.76%	65.42%	74.25%	75.61%	74.93%	60.38%	57.22%
Rat	77.01%	77.31%	77.01%	87.76%	100%	65.99%	75.15%	77.01%	62.79%	60.30%	59.72%
Pig	72.91%	72.91%	73.49%	65.42%	65.99%	100%	80.54%	81.79%	71.80%	63.58%	56.94%
Dog	83.53%	83.83%	83.83%	74.25%	75.15%	80.54%	100%	90.75%	72.38%	67.09%	63.06%
Cat	85.67%	85.67%	85.37%	75.61%	77.01%	81.79%	90.75%	100%	73.55%	66.13%	63.61%
Cow	68.60%	68.31%	68.31%	74.93%	62.79%	71.80%	72.38%	73.55%	100%	68.37%	64.69%
Opossum	66.13%	66.13%	66.77%	60.38%	60.30%	63.58%	67.09%	66.13%	68.37%	100%	53.89%
Platypus	61.94%	62.22%	62.22%	57.22%	59.72%	56.94%	63.06%	63.61%	64.69%	53.89%	100%

FAM124A	Human	Chimpanzee	Orangutan	Mouse	Rat	Pig	Dog	Cat	Cow	Opossum	Platypus
Human	100%	99.66%	98.72%	73.13%	73.13%	71.33%	85.83%	79.52%	75%	61.86%	70.68%
Chimpanzee	99.66%	100%	98.35%	72.95%	73.13%	71.33%	85.83%	79.15%	75%	61.68%	70.68%
Orangutan	98.72%	98.35%	100%	73.51%	73.32%	70.45%	85.02%	79.70%	75.73%	62.23%	70.30%
Mouse	73.13%	72.95%	73.51%	100%	92.91%	60.84%	83.81%	68.27%	63.79%	58.94%	71.43%
Rat	73.13%	73.13%	73.32%	92.91%	100%	60.84%	84.62%	68.63%	63.42%	58.76%	71.43%
Pig	71.33%	71.33%	70.45%	60.84%	60.84%	100%	85.02%	75.09%	75.37%	57.66%	73.31%
Dog	85.83%	85.83%	85.02%	83.81%	84.62%	85.02%	100%	83.81%	81.38%	59.67%	74.44%
Cat	79.52%	79.15%	79.70%	68.27%	68.63%	75.09%	83.81%	100%	75.92%	61.86%	72.56%
Cow	75%	75%	75.73%	63.79%	63.42%	75.37%	81.38%	75.92%	100%	58.39%	70.68%
Opossum	61.86%	61.68%	62.23%	58.94%	58.76%	57.66%	59.67%	61.86%	58.39%	100%	79.32%
Platypus	70.68%	70.68%	70.30%	71.43%	71.43%	73.31%	74.44%	72.56%	70.68%	79.32%	100%

PRMT2	Human	Chimpanzee	Orangutan	Mouse	Rat	Pig	Dog	Cat	Cow	Opossum	Platypus
Human	100%	99.77%	98.85%	79.79%	85.84%	81.46%	87.56%	88.02%	74.38%	N/A	68.67%
Chimpanzee	99.77%	100%	99.08%	80%	86.07%	81.22%	87.79%	88.25%	74.17%	N/A	68.89%
Orangutan	98.85%	99.08%	100%	80.42%	86.52%	81.69%	88.02%	88.44%	74.58%	N/A	68.89%
Mouse	79.79%	80%	80.42%	100%	96.40%	80.52%	85.48%	84.33%	75.42%	N/A	69.78%
Rat	85.84%	86.07%	86.52%	96.40%	100%	80.05%	85.25%	84.10%	75.21%	N/A	69.33%
Pig	81.46%	81.22%	81.69%	80.52%	80.05%	100%	83.18%	81.80%	76.88%	N/A	64.67%
Dog	87.56%	87.79%	88.02%	85.48%	85.25%	83.18%	100%	95.39%	78.54%	N/A	70.44%
Cat	88.02%	88.25%	88.44%	84.33%	84.10%	81.80%	95.39%	100%	77.50%	N/A	70.89%
Cow	74.38%	74.17%	74.58%	75.42%	75.21%	76.88%	78.54%	77.50%	100%	N/A	68.44%
Opossum	N/A	N/A	N/A	N/A	N/A	N/A	N/A	N/A	N/A	N/A	N/A
Platypus	68.67%	68.89%	68.89%	69.78%	69.33%	64.67%	70.44%	70.89%	68.44%	N/A	100%

AOC3	Human	Chimpanzee	Orangutan	Mouse	Rat	Pig	Dog	Cat	Cow	Opossum	Platypus
Human	100%	99.08%	97.25%	83.01%	82.44%	83.38%	88.40%	87.94%	81.96%	N/A	N/A
Chimpanzee	99.08%	100%	97.12%	82.88%	82.57%	83.24%	88.53%	88.07%	81.57%	N/A	N/A
Orangutan	97.25%	97.12%	100%	82.22%	81.78%	84.96%	87.22%	86.89%	81.19%	N/A	N/A
Mouse	83.01%	82.88%	82.22%	100%	93.58%	81.53%	83.18%	83.36%	79.25%	N/A	N/A
Rat	82.44%	82.57%	81.78%	93.58%	100%	80.21%	82.27%	83.09%	78.35%	N/A	N/A
Pig	83.38%	83.24%	84.96%	81.53%	80.21%	100%	85.92%	84.80%	81.96%	N/A	N/A
Dog	88.40%	88.53%	87.22%	83.18%	82.27%	85.92%	100%	92.14%	82.60%	N/A	N/A
Cat	87.94%	88.07%	86.89%	83.36%	83.09%	84.80%	92.14%	100%	81.19%	N/A	N/A
Cow	81.96%	81.57%	81.19%	79.25%	78.35%	81.96%	82.60%	81.19%	100%	N/A	N/A
Opossum	N/A	N/A	N/A	N/A	N/A	N/A	N/A	N/A	N/A	N/A	N/A
Platypus	N/A	N/A	N/A	N/A	N/A	N/A	N/A	N/A	N/A	N/A	N/A

EPX	Human	Chimpanzee	Orangutan	Mouse	Rat	Pig	Dog	Cat	Cow	Opossum	Platypus
Human	100%	99.58%	98.84%	89.11%	88.25%	85.31%	N/A	60.49%	N/A	80.09%	79.16%
Chimpanzee	99.58%	100%	99.03%	89.25%	88.39%	85.45%	N/A	60.49%	N/A	79.94%	79.16%
Orangutan	98.84%	99.03%	100%	65.92%	65.31%	62.43%	N/A	60.76%	N/A	66.05%	79.16%
Mouse	89.11%	89.25%	65.92%	100%	92.87%	84.46%	N/A	59.67%	N/A	81.02%	77.17%
Rat	88.25%	88.39%	65.31%	92.87%	100%	83.33%	N/A	58.86%	N/A	81.02%	76.92%
Pig	85.31%	85.45%	62.43%	84.46%	83.33%	100%	N/A	56.95%	N/A	75.93%	76.43%
Dog	N/A	N/A	N/A	N/A	N/A	N/A	N/A	N/A	N/A	N/A	N/A
Cat	60.49%	60.49%	60.76%	59.67%	58.86%	56.95%	N/A	100%	N/A	30.40%	49.38%
Cow	N/A	N/A	N/A	N/A	N/A	N/A	N/A	N/A	N/A	N/A	N/A
Opossum	80.09%	79.94%	66.05%	81.02%	81.02%	75.93%	N/A	30.40%	N/A	100%	78.16%
Platypus	79.16%	79.16%	79.16%	77.17%	76.92%	76.43%	N/A	49.38%	N/A	78.16%	100%

GZMM	Human	Chimpanzee	Orangutan	Mouse	Rat	Pig	Dog	Cat	Cow	Opossum	Platypus
Human	100%	96.89%	88.40%	64.77%	63.64%	62.31%	N/A	N/A	63.95%	45.79%	49.48%
Chimpanzee	96.89%	100%	87.60%	65.53%	64.04%	62.69%	N/A	N/A	64.73%	46.15%	49.48%
Orangutan	88.40%	87.60%	100%	57.58%	56.06%	58.46%	N/A	N/A	60.47%	43.96%	49.48%
Mouse	64.77%	65.53%	57.58%	100%	86.74%	61.54%	N/A	N/A	62.79%	48.35%	44.33%
Rat	63.64%	64.04%	56.06%	86.74%	100%	60.77%	N/A	N/A	62.79%	47.99%	43.30%
Pig	62.31%	62.69%	58.46%	61.54%	60.77%	100%	N/A	N/A	70.54%	45.79%	43.30%
Dog	N/A	N/A	N/A	N/A	N/A	N/A	N/A	N/A	N/A	N/A	N/A
Cat	N/A	N/A	N/A	N/A	N/A	N/A	N/A	N/A	N/A	N/A	N/A
Cow	63.95%	64.73%	60.47%	62.79%	62.79%	70.54%	N/A	N/A	100%	46.15%	43.30%
Opossum	45.79%	46.15%	43.96%	48.35%	47.99%	45.79%	N/A	N/A	46.15%	100%	47.42%
Platypus	49.48%	49.48%	49.48%	44.33%	43.30%	43.30%	N/A	N/A	43.30%	47.42%	100%

HRC	Human	Chimpanzee	Orangutan	Mouse	Rat	Pig	Dog	Cat	Cow	Opossum	Platypus
Human	100%	96.25%	78.28%	49.73%	46.42%	69.03%	57.10%	N/A	51.29%	N/A	35.69%
Chimpanzee	96.25%	100%	77.02%	48.24%	44.83%	66.94%	56.97%	N/A	49.83%	N/A	35.69%
Orangutan	78.28%	77.02%	100%	32.66%	30.24%	41.13%	44.22%	N/A	36.11%	N/A	21.85%
Mouse	49.73%	48.24%	32.66%	100%	73.74%	53.39%	45.55%	N/A	41.17%	N/A	33.54%
Rat	46.42%	44.83%	30.24%	73.74%	100%	52.74%	43.03%	N/A	40.61%	N/A	32.00%
Pig	69.03%	66.94%	41.13%	53.39%	52.74%	100%	51.26%	N/A	47.24%	N/A	31.08%
Dog	57.10%	56.97%	44.22%	45.55%	43.03%	51.26%	100%	N/A	49.16%	N/A	29.54%
Cat	N/A	N/A	N/A	N/A	N/A	N/A	N/A	N/A	N/A	N/A	N/A
Cow	51.29%	49.83%	36.11%	41.17%	40.61%	47.24%	49.16%	N/A	100%	N/A	34.15%
Opossum	N/A	N/A	N/A	N/A	N/A	N/A	N/A	N/A	N/A	N/A	N/A
Platypus	35.69%	35.69%	21.85%	33.54%	32.00%	31.08%	29.54%	N/A	34.15%	N/A	100%

ADIRF	Human	Chimpanzee	Orangutan	Mouse	Rat	Pig	Dog	Cat	Cow	Opossum	Platypus
Human	100%	100%	98.68%	N/A	N/A	92%	N/A	84.21%	N/A	84.21%	N/A
Chimpanzee	100%	100%	98.68%	N/A	N/A	92%	N/A	84.21%	N/A	84.21%	N/A
Orangutan	98.68%	98.68%	100%	N/A	N/A	90.67%	N/A	82.89%	N/A	82.89%	N/A
Mouse	N/A	N/A	N/A	N/A	N/A	N/A	N/A	N/A	N/A	N/A	N/A
Rat	N/A	N/A	N/A	N/A	N/A	N/A	N/A	N/A	N/A	N/A	N/A
Pig	92%	92%	90.67%	N/A	N/A	100%	N/A	89.47%	N/A	63.16%	N/A
Dog	N/A	N/A	N/A	N/A	N/A	N/A	N/A	N/A	N/A	N/A	N/A
Cat	84.21%	84.21%	82.89%	N/A	N/A	89.47%	N/A	100%	N/A	60.53%	N/A
Cow	N/A	N/A	N/A	N/A	N/A	N/A	N/A	N/A	N/A	N/A	N/A
Opossum	65.79%	65.79%	64.47%	N/A	N/A	63.16%	N/A	60.53%	N/A	100%	N/A
Platypus	N/A	N/A	N/A	N/A	N/A	N/A	N/A	N/A	N/A	N/A	N/A

S2 Fig. VSMC orthologue sequence conservation between 11 vertebrate species. The sequence identity for orthologues for specific VSMC proteins was determined using the Ensembl database. Please refer to the main text for detailed methods. In brief, the query species used for the analysis is listed in the first column of each array; the targets are listed on the first row of each array. Nine genes on the left side of the figure were chosen to illustrate the diversity of conservation of VSMC proteins, including some proteins discovered in this study and others considered cover VSMC markers (eg. ACTA2). The 18 remaining genes in the center and on the right side of the fugure correspond to those chose for immunohistochemical analysis in Fig 1-2; the analysis of these 18 factors used the best (most similar to human) orthologue as quesry for searches in order to avoid over-reporting of divergence (as described in the methods). The 27 arrays of data were used to generate the heatmaps in Fig 6. ARHGEF35 was chosen for display because it is a human protein that by multiple database analyses failed to display a non-human orthologue; however, it was not listed in Table 1 because data was missing from GeneCards. N/A is listed when no orthologue was present in the indicated species.
