# Supplementary material for: Tripartite factors leading to molecular divergence between human and murine smooth muscle
Source: PLoS One. 2020 Jan 16;15(1):e0227672. doi: 10.1371/journal.pone.0227672 (PMC6964862; doi:10.1371/journal.pone.0227672)
Supplement: S3 Fig — (PDF) [file pone.0227672.s003.pdf]

Gene	No Close Orthologue in
A2M	platypus, opossum
ALG1L*	mouse, rat, pig, dog, cat, cow, orangutan, opossum, platypus
ARGFX*	mouse, rat, pig, dog, cat, cow, opossum, platypus
AVPI1	platypus
C9orf47	platypus
C9orf75*	mouse, rat, pig, dog, cat, cow, opossum, platypus
C9orf152*	mouse, rat, dog, cow, opossum, platypus
C12orf49	opossum, platypus
C17orf78*	mouse, rat, pig, cat, cow, opossum, platypus
C19orf60	rat, dog, opossum, platypus
CABP7	opossum, platypus
CCDC71	cat, orangutan, opossum, platypus
CCDC140*	mouse, rat, pig, dog, cat, cow, orangutan, opossum, platypus
CRB3*	mouse, rat, dog, cow, opossum, platypus
CRYBA2	opossum, platypus
CT47A11*	mouse, rat, pig, dog, cat, cow, opossum, platypus
DMRTC1B*	mouse, rat, pig, dog, cat, cow, opossum, platypus
FAM100B	dog, platypus
GIMAP1*	mouse, rat, pig, dog, cat, cow, chimpanzee, opossum, platypus
GPER	chimpanzee, orangutan
GRRP1	opossum, platypus
HRC*	mouse, rat, dog, cat, cow, opossum, platypus
IFNA2*	mouse, rat, pig, dog, cat, cow, opossum, platypus
JMJD4	cat, opossum, platypus
LDLRAD2*	mouse, rat, pig, opossum, platypus
LMTK3	rat, cat, opossum
LONRF1	opossum
MAP7D3*	mouse, rat, pig, dog, cat, cow, opossum, platypus
MAS1L*	mouse, rat, pig, dog, cat, cow, chimpanzee, opossum, platypus
MYADML2	platypus
NAB1	platypus
NES*	mouse, rat, pig, dog, cat, cow, opossum, platypus
NMB*	mouse, rat, dog, opossum, platypus
PNRC2	dog, cat, chimpanzee, platypus
PODXL*	mouse, rat, pig, dog, cat, cow, opossum, platypus
RAPSN	pig, opossum
RLN3	dog, opossum, platypus
S100A10	platypus
SCNM1	platypus
SDPR	platypus
SNAP47*	mouse, cat, opossum, platypus
SPANXA1*	mouse, rat, pig, dog, cat, cow, orangutan, opossum, platypus
SYNPO2L	platypus
TAPBP	opossum, platypus
TIAF1*	mouse, rat, pig, dog, cat, cow, chimpanzee, orangutan, opossum, platypus
TRIM59	chimpanzee, orangutan, opossum, platypus
TRIM73*	mouse, rat, pig, dog, cat, cow, opossum, platypus
TSPYL1*	mouse, rat, pig, dog, opossum, platypus
ULBP2*	mouse, rat, pig, dog, cat, cow, orangutan, opossum, platypus
VAMP5	orangutan, opossum, platypus
ZBTB5	platypus
ZC3HAV1L	cat, opossum, platypus
ZNF527*	mouse, rat, opossum, platypus
ZNF7*	mouse, opossum, platypus

S3 Fig. Absence of vascular orthologues in diverse vertebrate species. We list the 54 human vascular proteins that did not have a close orthologue or absent in at least one species. For each protein, we specifiy the species that did not have an orthologue annotated in our multiple-database search. TIAF1 is shown in red because it was a human only protien (absent in 10 other vertebrate species). Asterisk denote factors that were absent in mouse.
