# Supplementary material for: Tripartite factors leading to molecular divergence between human and murine smooth muscle
Source: PLoS One. 2020 Jan 16;15(1):e0227672. doi: 10.1371/journal.pone.0227672 (PMC6964862; doi:10.1371/journal.pone.0227672)
Supplement: S4 Fig — (PDF) [file pone.0227672.s004.pdf]

Chimpanzee (Pan Troglodytes)

All proteins	VSMC or VSMC/EC
GIMAP1*	GPER*
GPER*	MAS1L*
MAS1L*	PNRC2*
PNRC2*	TIAF1*
TIAF1*	
TRIM59*	

Orangutan (Pongo Abelii)

All proteins	VSMC or VSMC/EC
ALG1L*	ALG1L*
CCDC71*	CCDC140*
CCDC140*	GPER*
GPER*	SPANXA1*
SPANXA1*	TIAF1*
TIAF1*	ULBP2*
TRIM59*	
ULBP2*	
VAMP5	

Mouse (Mus musculus)

All proteins	VSMC or VSMC/EC
ARGFX*	ARGFX*
ALG1L	ALG1L
C9orf75	C9orf75
C9orf152	C9orf152
C17orf78	C17orf78
CCDC140*	CCDC140*
CRB3	CRB3
CT47A11*	CT47A11*
DMRTC1B*	DMRTC1B*
GIMAP1	GIMAP1
HRC	HRC
IFNA2	IFNA2
LDLRAD2*	LDLRAD2*
MAP7D3	MAP7D3
MAS1L*	MAS1L*
NES	NES
NMB	NMB
PODXL	PODXL
SNAP47	SNAP47
SPANXA1*	SPANXA1*
TIAF1*	TIAF1*
TRIM73*	TSPYL1
TSPYL1	ULBP2
ULBP2	ZNF7
ZNF7	ZNF527*
ZNF527*	

Rat (Rattus Norvegicus)

All proteins	VSMC or VSMC/EC
ARGFX*	ARGFX*
ALG1L	ALG1L
C9orf75	C9orf75
C9orf152	C9orf152
C17orf78	C17orf78
CCDC140*	CCDC140*
C19orf60	C19orf60
CRB3	CRB3
CT47A11*	CT47A11*
DMRTC1B*	DMRTC1B*
GIMAP1	GIMAP1
HRC	HRC
IFNA2	IFNA2
LDLRAD2*	LDLRAD2*
LMTK3*	LMTK3*
MAP7D3	MAP7D3
MAS1L*	MAS1L*
NES	NES
NMB	NMB
PODXL	PODXL
SPANXA1*	SPANXA1*
TIAF1*	TIAF1*
TRIM73*	TSPYL1
TSPYL1	ULBP2
ULBP2	ZNF527*
ZNF527*	

Pig (Sus scrofa)

All proteins	VSMC or VSMC/EC
ALG1L	ALG1L
ARGFX	ARGFX
C9orf75	C9orf75
C17orf78	C17orf78
CCDC140*	CCDC140*
CT47A11*	CT47A11*
DMRTC1B*	DMRTC1B*
GIMAP1	GIMAP1
IFNA2	IFNA2
LDLRAD2*	LDLRAD2*
MAP7D3	MAP7D3
MAS1L	MAS1L
NES	NES
PODXL	PODXL
RAPSN*	RAPSN*
SPANXA1*	SPANXA1*
TIAF1*	TIAF1*
TRIM73*	TSPYL1
TSPYL1	ULBP2
ULBP2	

Dog (Canis lupus familiaris)

All proteins	VSMC or VSMC/EC
ALG1L	ALG1L
ARGFX*	ARGFX*
C9orf75	C9orf75
C9orf152	C9orf152
C19orf60	C19orf60
CCDC140*	CCDC140*
CRB3*	CT47A11
CT47A11	DMRTC1B*
DMRTC1B*	GIMAP1
FAM100B*	HRC
GIMAP1	IFNA2*
HRC	MAP7D3
IFNA2*	MAS1L
MAP7D3	NES
MAS1L	NMB
NES	PNRC2
NMB	PODXL
PNRC2	SPANXA1*
PODXL	TIAF1*
RLN3*	TSPYL1
SPANXA1*	ULBP2
TIAF1*	
TRIM73*	
TSPYL1	
ULBP2	

Cat (Felis Catus)

All proteins	VSMC or VSMC/EC
ALG1L	ALG1L
ARGFX*	ARGFX*
C9orf75*	C9orf75*
C17orf78	C17orf78
CCDC71*	CCDC140*
CCDC140*	CT47A11*
CT47A11*	DMRTC1B*
DMRTC1B*	GIMAP1
GIMAP1	HRC*
HRC*	IFNA2*
IFNA2*	JMJD4
JMJD4	LMTK3*
LMTK3*	MAP7D3
MAP7D3	MAS1L*
MAS1L*	NES*
NES*	PNRC2*
PNRC2*	PODXL
PODXL	SNAP47
SNAP47	SPANXA1*
SPANXA1*	TIAF1*
TIAF1*	ULBP2*
TRIM73*	ZC3HAV1L
ULBP2*	
ZC3HAV1L	

Cow (Bos Taurus)

All proteins	VSMC or VSMC/EC
ALG1L	ALG1L
ARGFX	ARGFX
C9orf75	C9orf75
C9orf152	C9orf152
CCDC140*	CCDC140*
CRB3*	C17orf78
C17orf78	CT47A11
CT47A11	DMRTC1B*
DMRTC1B*	GIMAP1
GIMAP1	HRC
HRC	IFNA2
IFNA2	MAP7D3
MAP7D3	MAS1L
MAS1L	NES
NES	PODXL
PODXL	SPANXA1*
SPANXA1*	TIAF1*
TIAF1*	ULBP2
TRIM73*	
ULBP2	

Oppossum (Monodelphis domestica)

All proteins	VSMC or VSMC/EC
A2M	ALG1L
ALG1L	ARGFX*
ARGFX*	C9orf75
C9orf75	C9orf152*
C9orf152*	C17orf78
C12orf49	C19orf60*
C17orf78	CCDC140*
C19orf60*	CT47A11*
CABP7	DMRTC1B*
CCDC71	GIMAP1
CCDC140*	HRC*
CRB3*	IFNA2*
CRYBA2*	JMJD4
CT47A11*	LDLRAD2
DMRTC1B*	LMTK3*
GIMAP1	MAP7D3
GRRP1*	MAS1L
HRC*	NES
IFNA2*	NMB
JMJD4	PODXL
LDLRAD2	RAPSN*
LMTK3*	SNAP47
LONRF1*	SPANXA1*
MAP7D3	TIAF1*
MAS1L	TSPYL1*
NES	ULBP2
NMB	ZC3HAV1L
PODXL	ZNF527*
RLN3*	ZNF7*
RAPSN*	
SNAP47	
SPANXA1*	
TAPBP	
TIAF1*	
TRIM59	
TRIM73*	
TSPYL1*	
ULBP2	
VAMP5*	
ZC3HAV1L	
ZNF527*	
ZNF7*	

Platypus (Ornithorhynchus anatinus)

All proteins	VSMC or VSMC/EC
A2M	ALG1L*
ALG1L*	ARGFX*
ARGFX*	AVPI1*
AVPI1*	C9orf47*
C9orf47*	C9orf75*
C9orf75*	C9orf152*
C9orf152*	C17orf78*
C12orf49*	C19orf60*
C17orf78*	CCDC140*
C19orf60*	CT47A11*
CABP7*	DMRTC1B*
CCDC71	HRC
CCDC140*	IFNA2*
CRB3*	JMJD4*
CRYBA2*	LDLRAD2*
CT47A11*	MAP7D3
DMRTC1B*	MAS1L*
FAM100B*	MYADML2*
GIMAP1*	NAB1*
GRRP1*	NES*
HRC	NMB*
IFNA2*	PNRC2*
JMJD4*	SNAP47*
LDLRAD2*	SPANXA1*
MAP7D3	SYNPO2L
MAS1L*	TIAF1*
MYADML2*	TSPYL1*
NAB1*	ULBP2*
NES*	ZC3HAV1L*
NMB*	ZNF527*
PNRC2*	ZNF7*
PODXL*	
RLN3	
S100A10*	
SCNM1*	
SDPR*	
SNAP47*	
SPANXA1*	
SYNPO2L	
TAPBP*	
TIAF1*	
TRIM59	
TRIM73*	
TSPYL1*	
ULBP2*	
VAMP5	
ZBTB5*	
ZC3HAV1L*	
ZNF527*	
ZNF7*	

S4 Fig. Absence of vascular orthologues in specific vertebrate species. We show the human factors (from among 54 non-uniformaly conserved protiens) that do not have orthologues in each species displayed. The lists are further divided by vascular cell type of expression, with factors that are expressed in VSMC on the right column of each group. Yellow highlighting indicates EC proteins. Note that a majority of non-conserved proteins are found in VSMC in every species show. Asterisk denote factors that were absent in mouse.
