# Supplementary material for: Tripartite factors leading to molecular divergence between human and murine smooth muscle
Source: PLoS One. 2020 Jan 16;15(1):e0227672. doi: 10.1371/journal.pone.0227672 (PMC6964862; doi:10.1371/journal.pone.0227672)
Supplement: S5 Fig — (PDF) [file pone.0227672.s005.pdf]

**Human Specific**	TIAF1	ARHGEF35*
**Mouse but not rat**	C19orf60	LMTK3
**Rat but not mouse**	SNAP47	ZNF7
**Neither mouse nor rat**	ALG1L	LDLRAD2
	ARGFX	MAP7D3
	C9orf75	MAS1L
	C9orf152	NES
	C17orf78	NMB
	CCDC140	PODXL
	CRB3	SPANXA1
	CT47A11	TIAF1
	DMRTC1B	TRIM73
	GIMAP1	TSPYL1
	HRC	ULBP2
	IFNA2	ZNF527
**Primate specific**	ALG1L	MAP7D3
	ARGFX	MAS1L
	C9orf75	NES
	CCDC140	PODXL
	CT47A11	SPANXA1
	DMRTC1B	TRIM73
	GIMAP1	ULBP2
	IFNA2	
**Chimp but not orangutan**	ALG1L	SPANXA1
	CCDC71	ULBP2
	CCDC140	VAMP5
**Orangutan but not chimp**	GIMAP1	PNRC2
	MAS1L	
**Neither chimp nor orangutan**	GPER	TRIM59

S5 Fig. Human proteins that lack orthologues in rodent and non-human primates. We list proteins (from among the core list of 54 non-uniformly conserved vascular molecules) that are absent from mouse, rat, chimp, and/or orangutan. Primate analysis was limited to chimp and orangutan. We included ARHGEF35 which was absent from all non-human species in multiple database analysis but not included in the list of 54 non-uniformly conserved proteins since data was missing from GeneCards.
