# Supplementary material for: Tripartite factors leading to molecular divergence between human and murine smooth muscle
Source: PLoS One. 2020 Jan 16;15(1):e0227672. doi: 10.1371/journal.pone.0227672 (PMC6964862; doi:10.1371/journal.pone.0227672)
Supplement: S6 Fig — (PDF) [file pone.0227672.s006.pdf]

	FVB/N	C57/BL6	Differences between strains
ADIRF	Y	N	N/A
AGXT	Y	Y	N
AOC3	Y	Y	N
DES	Y	Y	N
EPX	Y	Y	N
FAM124A	Y	Y	N
GPX8	Y	Y	N
GZMM	Y	Y	N
HRC	Y	Y	N
LPHN2(=ADGRL2)	Y	Y	N
LRRC41	Y	Y	N
MTFR1	Y	N	N/A
NEURL4	Y	N	N/A
PRMT2	Y	N	N/A
RHOF	Y	Y	N
ST6GALNAC6	Y	Y	N
TBC1D2B	Y	N	N/A
TEX261	Y	N	N/A

S6 Fig. Mouse strains tested in immunohistochemical comparison to human tissues. In Fugures 1 and 2, we show immunohistochemical analysis of mouse tissues using 18 antibodies, as described in methods. The background strains used for the analysis of each protein are shown. We did not note any strain-to-strain differences for any of the proteins that were analyzed in two strains.
