# Supplementary material for: Tripartite factors leading to molecular divergence between human and murine smooth muscle
Source: PLoS One. 2020 Jan 16;15(1):e0227672. doi: 10.1371/journal.pone.0227672 (PMC6964862; doi:10.1371/journal.pone.0227672)
Supplement: S7 Fig — (PDF) [file pone.0227672.s007.pdf]

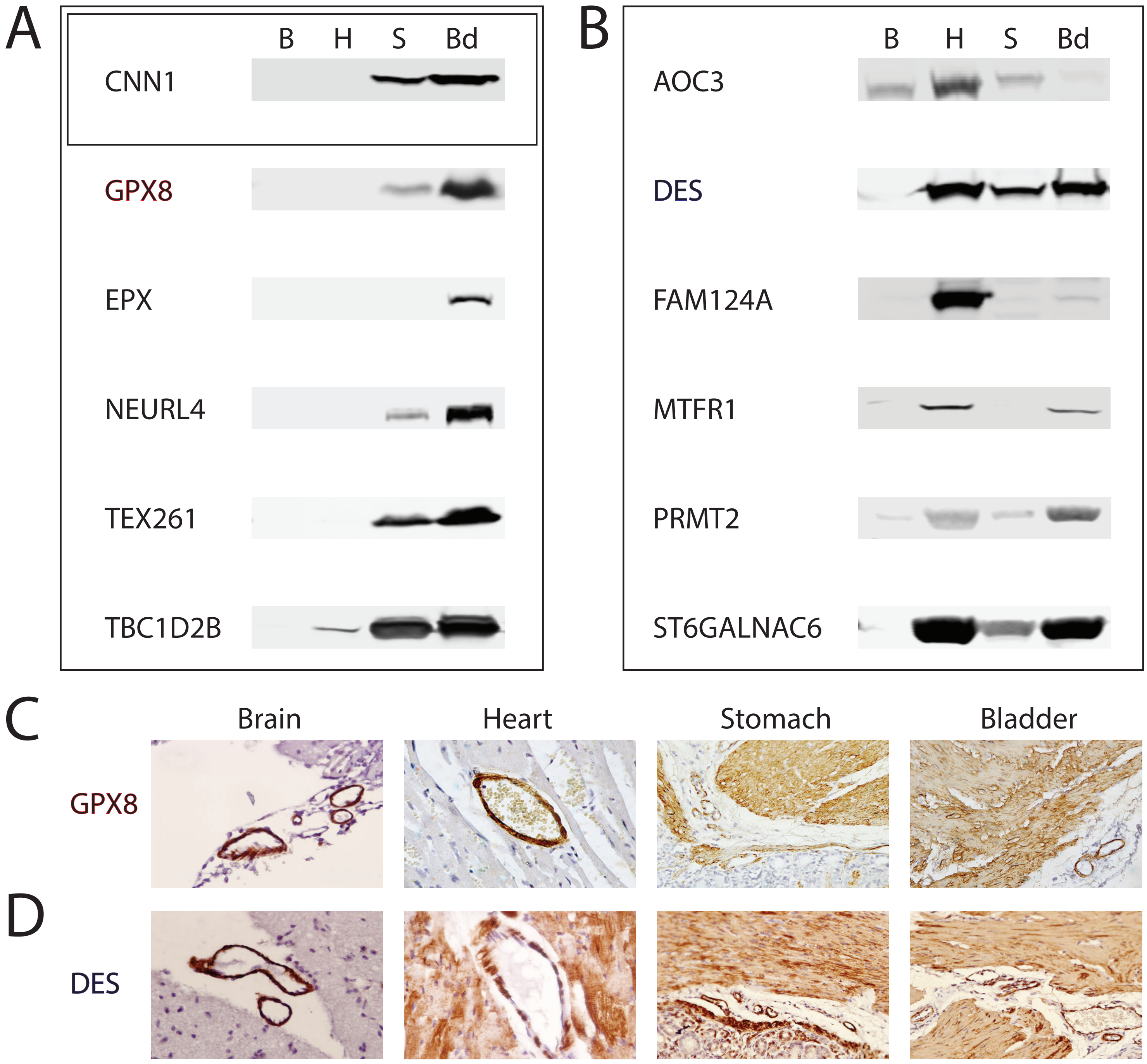

S7 Fig. Expression of each protein in mouse brain, heart, stomach, and Bladder. Fractions of mouse tissue lysates were analyzed for presence of indicated proteins. CNN1, a canonical marker for SMC protein, is showing in inner box of panel A and examples of similar expression pattern are on the same panel. Stomach and Bladder which have higher SMC content compared to brain showed higher expression pattern on WB and panel C shows the representative IHC example of GPX8 from this group of protein. Proteins who shows higher expression pattern on Heart compare to Brain are showing in panel B and panel D shows the representative IHC example of DES from these lists. Results were reproduced at least three times.
