# Supplementary material for: Tripartite factors leading to molecular divergence between human and murine smooth muscle
Source: PLoS One. 2020 Jan 16;15(1):e0227672. doi: 10.1371/journal.pone.0227672 (PMC6964862; doi:10.1371/journal.pone.0227672)
Supplement: S8 Fig — (PDF) [file pone.0227672.s008.pdf]

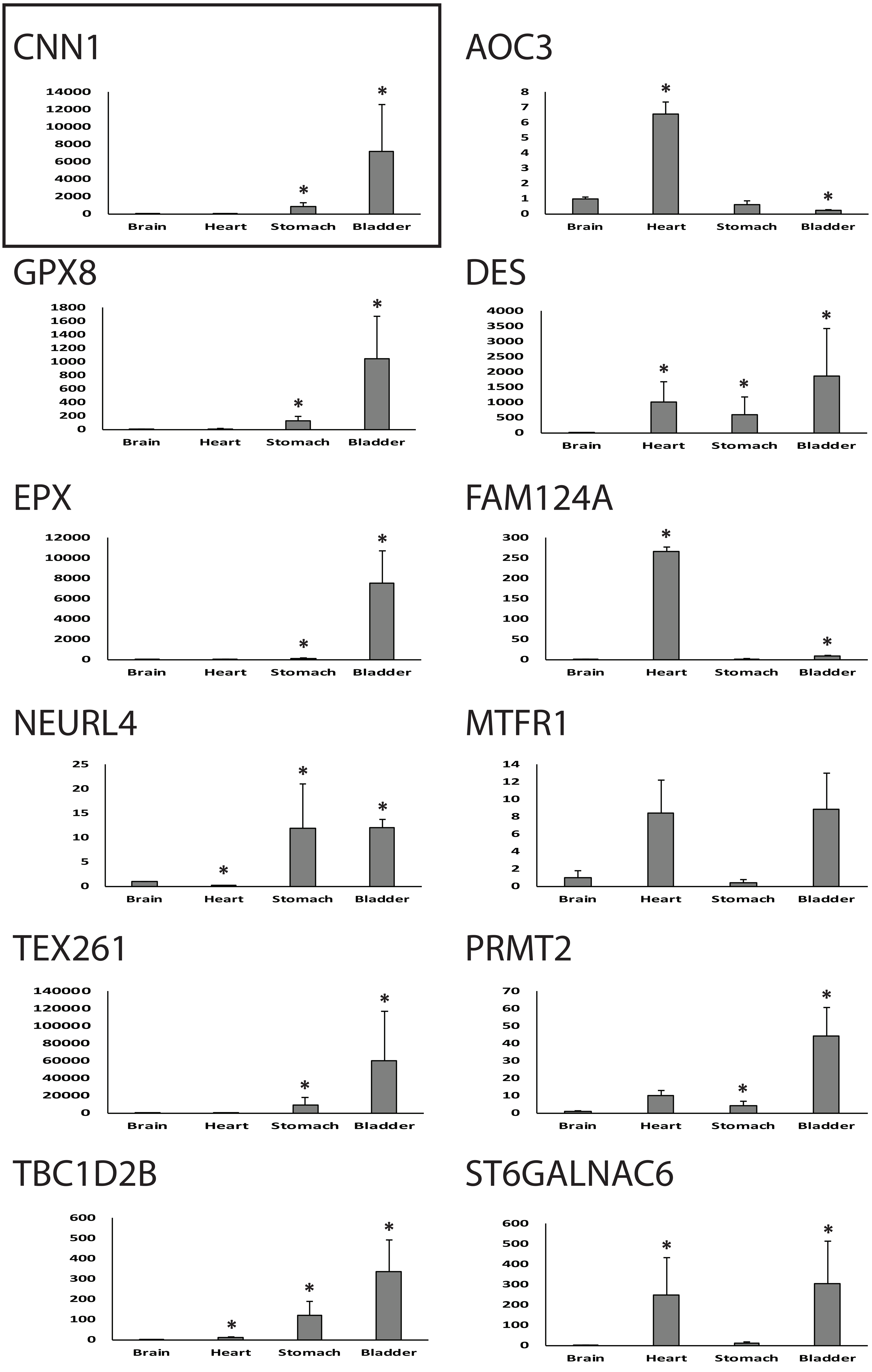

S8 Fig. Quantification of protein expression compared to brain. Each protein was normalized by total protein measured by Revert Total Protein Stain (Li-COR #926-11021) then T-test were performed with 2 tailed test with two sample equal variance. The graphs are showing the normalized value to Brain and significance was considered $p<0.05$ and indicated with * mark.
